# Combined PARP and ATR inhibition potentiates genome instability and cell death in ATM-deficient cancer cells

**DOI:** 10.1038/s41388-020-1328-y

**Published:** 2020-05-23

**Authors:** Rebecca L. Lloyd, Paul W. G. Wijnhoven, Antonio Ramos-Montoya, Zena Wilson, Giuditta Illuzzi, Katarzyna Falenta, Gemma N. Jones, Neil James, Christophe D. Chabbert, Jonathan Stott, Emma Dean, Alan Lau, Lucy A. Young

**Affiliations:** 1 Bioscience, Oncology R&D, AstraZeneca, Cambridge, UK; 2 The Wellcome trust and CRUK Gurdon Institute, and Department of Biochemistry, University of Cambridge, Cambridge, UK; 3 Bioscience, Oncology R&D, AstraZeneca, Alderley Park, UK; 4 Translational Medicine, Oncology R&D, AstraZeneca, Cambridge, UK; 5 Quantitative Biology, Discovery Science, Oncology R&D, AstraZeneca, Cambridge, UK; 6 Research and Early Development, Oncology R&D, AstraZeneca, Cambridge, UK

**Keywords:** Drug development, Targeted therapies, Predictive markers, Predictive markers

## Abstract

The poly (ADP-ribose) polymerase (PARP) inhibitor olaparib is FDA approved for the treatment of *BRCA*-mutated breast, ovarian and pancreatic cancers. Olaparib inhibits PARP1/2 enzymatic activity and traps PARP1 on DNA at single-strand breaks, leading to replication-induced DNA damage that requires BRCA1/2-dependent homologous recombination repair. Moreover, DNA damage response pathways mediated by the ataxia-telangiectasia mutated (ATM) and ataxia-telangiectasia mutated and Rad3-related (ATR) kinases are hypothesised to be important survival pathways in response to PARP-inhibitor treatment. Here, we show that olaparib combines synergistically with the ATR-inhibitor AZD6738 (ceralasertib), in vitro, leading to selective cell death in ATM-deficient cells. We observe that 24 h olaparib treatment causes cells to accumulate in G2-M of the cell cycle, however, co-administration with AZD6738 releases the olaparib-treated cells from G2 arrest. Selectively in *ATM*-knockout cells, we show that combined olaparib/AZD6738 treatment induces more chromosomal aberrations and achieves this at lower concentrations and earlier treatment time-points than either monotherapy. Furthermore, single-agent olaparib efficacy in vitro requires PARP inhibition throughout multiple rounds of replication. Here, we demonstrate in several ATM-deficient cell lines that the olaparib and AZD6738 combination induces cell death within 1–2 cell divisions, suggesting that combined treatment could circumvent the need for prolonged drug exposure. Finally, we demonstrate in vivo combination activity of olaparib and AZD6738 in xenograft and PDX mouse models with complete ATM loss. Collectively, these data provide a mechanistic understanding of combined PARP and ATR inhibition in ATM-deficient models, and support the clinical development of AZD6738 in combination with olaparib.

## Introduction

Inhibitors of core proteins involved in the DNA damage response (DDR) are being explored for their potential to selectively target cancer cells, such as by exploiting their high mutational burden and the principle of synthetic lethality [1–3]. For example, poly (ADP-ribose) polymerase (PARP) inhibitors are approved for the treatment of *BRCA-*mutated breast, ovarian and pancreatic cancers [4–8]. Although PARP1 is involved in various DDR-related processes, the current hypothesis explaining this selective toxicity is that olaparib inhibits PARP1 during DNA single-strand-break repair, and traps PARP1 onto DNA. Olaparib-induced DNA lesions can cause replication-induced DNA damage following collisions with, and collapse of, replication forks [9]. Such DNA lesions can only be faithfully repaired by homologous recombination (HR), rendering cancer cells deficient in BRCA1/2-dependent homologous recombination repair (HRR) highly sensitive to olaparib [4, 5]. Furthermore, clinical trials in patients with metastatic castration-resistant prostate cancer harbouring other HRR gene mutations have shown promising results [10, 11]. This suggests that PARP inhibitors could be expanded to other indications, including *BRCA-*mutated prostate cancer and those harbouring mutations in DDR proteins such as ataxia-telangiectasia mutated (ATM) [12–15]. ATM is also important for HRR signalling [16], however, the mechanism of olaparib-sensitivity in ATM-deficient cells differs from canonical HRR-deficiency, with ATM counteracting toxic end-joining of single-ended double-strand breaks (seDSBs) [17].

Inhibitors of other DDR factors are also in development, including those targeting ATM, ataxia-telangiectasia mutated and Rad3-related (ATR), and DNA-dependent protein kinase (DNA-PK). These are fundamental kinases involved in the detection, signalling and DNA repair pathway choice, alongside regulating DDR processes including DNA damage checkpoint activation, senescence and apoptosis [16]. Furthermore, control of DNA replication and the cell cycle are inherently linked to preserving genome stability and the DDR, leading to inhibitors targeting kinases such as WEE1 being developed. Despite ATR and ATM converging on many targets and functions [18–20], their activation occurs by different lesions, consistent with ATR and ATM inhibitors showing pre-clinical efficacy in different contexts. ATM and DNA-PK inhibitors are being explored as chemo- and radiosensitisers due to their fundamental role in DNA DSB repair [21, 22]. Conversely, ATR’s functions in regulating G2-M checkpoint activation, replication fork stability and late-origin firing [23] appear to dominate ATR-inhibitor efficacy, with ATR and WEE1 inhibitors showing efficacy in tumours with high replication stress [24–26]. ATM-deficient cells are also hypersensitive to ATR inhibition, at least partly due to a greater dependency on ATR for DNA repair and checkpoint control [27–29]. This presents a clinical opportunity as ATM inactivation has been reported in a high proportion of metastatic prostate, lung, haematological, gastric and colorectal tumours [11, 30, 31].

Emerging evidence also suggests that combining DDR inhibitors can enhance tumour killing and overcome acquired resistance. For example, combined ATR and PARP inhibition selectively re-sensitises olaparib-resistant BRCA1-deficient cells arisen through various mechanisms [32, 33]. Furthermore, it has been suggested that with optimised dose scheduling, combined PARP and WEE1 or ATR inhibition can enhance tumour killing with minimal systemic toxicity, owing to higher basal levels of replication stress in malignant versus normal tissue [34]. However, the full spectrum of possible efficacious combinations, and biomarkers that predict their tumour-selectivity, has not been fully explored.

In this study, we looked for drug-combination opportunities with olaparib by screening for tumour background-selective synergies with other DDR inhibitors. We show that olaparib combines synergistically with the ATR-inhibitor AZD6738 (ceralasertib) [35], leading to cell death in ATM-deficient backgrounds. We demonstrate that generation of mitosis-associated DNA damage and commitment to apoptosis occur earlier and at lower concentrations following combination treatment than with either single-agent, providing mechanistic insight and rationale for exploring combined PARP and ATR inhibition in the clinic.

## Results

### Olaparib synergises with ATR inhibition in ATM-deficient cancer cells

**Fig. 1:**
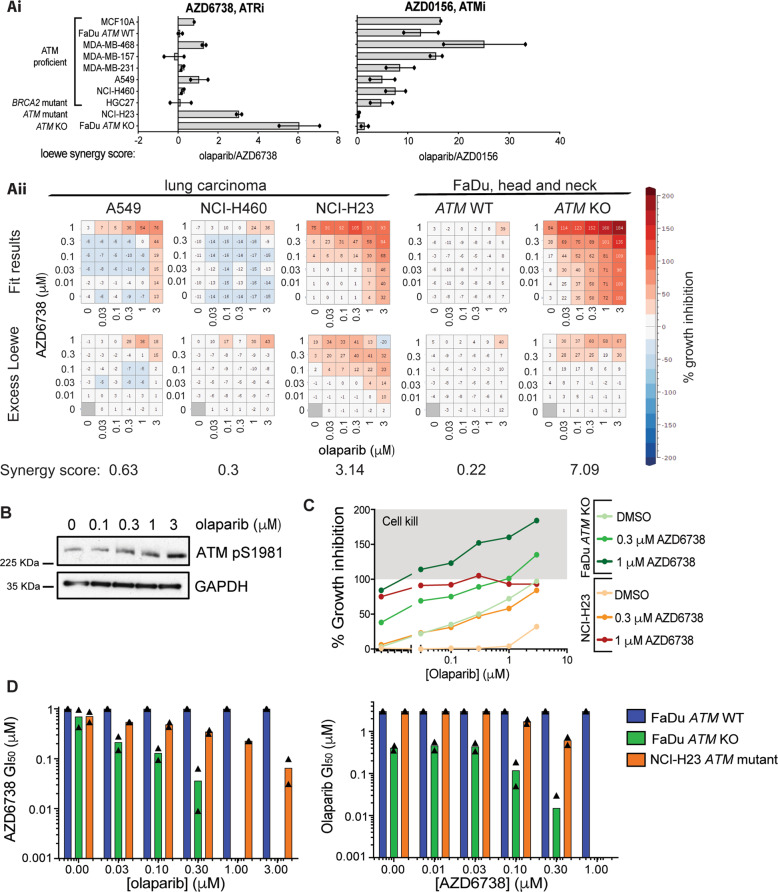
The ATR inhibitor (AZD6738) and olaparib are synergistic in ATM-deficient cell lines. **a** (i) Loewe synergy scores for olaparib in combination with the ATR (AZD6738) and ATM (AZD0156) kinase inhibitors across different cell lines. Cell viability was measured by a sytox green live-dead assay, and synergy scores calculated using the Loewe additivity model. Higher positive scores indicate greater synergistic activity. Error bars = Mean ± SEM (*n* = 2, excluding MCF10A). (ii) Representative 6 × 6 synergy matrix heatmaps for olaparib and AZD6738 treatment in ATM-deficient and -proficient FaDu and lung carcinoma cell lines. ‘Fitted results’ represent the growth inhibitory (0–100) and cytotoxic activity (100–200) based on curves fitted to the raw viability values. ‘Loewe excess’ represents the calculated excess activity above that expected from an additive combination, based on the Loewe additivity model. Loewe synergy scores are shown below the heatmaps. **b** Immunoblot for ATM activation via ATM pS1981 in FaDu *ATM*-WT cells following 24 h olaparib treatment. **c** Representative % growth inhibition curves for FaDu *ATM*-KO and NCI-H23 cells following single-agent and combination treatment with olaparib and AZD6738. Cytostatic effects are observed in the 0–100% range and cytotoxic effects between 100 and 200%. **d** GI_50_ values for olaparib and AZD6738 single-agent and combination treatments, determined by a sytox green live-dead assay (*n* = 2).

*BRCA1/2*-mutated tumours exhibit pronounced sensitivity to olaparib due to their inability to repair the DSBs that arise upon treatment [36]. We hypothesised that further to BRCA-dependent HRR, other DDR pathways could contribute to olaparib sensitivity. Therefore, identifying other genetic vulnerabilities to PARP inhibition, and/or inhibiting functional DDR pathways, could enhance PARP-inhibitor efficacy and expand their use to new patient populations. Previous work identified olaparib/DNAPKi (AZD7648) combination activity in ATM-deficient cells [22]. Therefore, we assessed the cytotoxicity of olaparib in combination with inhibitors against ATM (AZD0156), ATR (AZD6738) and WEE1 (AZD1775), across a panel of cell lines to identify synergistic combinations selective for genetic backgrounds including those with HRR- and ATM deficiencies (Fig. 1a and Supplementary Fig. 1A).

Olaparib was more cytotoxic in *BRCA*-mutated cells (MDA-MB-436, HGC-27) and those deficient in ATM signalling (FaDu; *ATM*-knockout (KO), NCI-H23; *ATM* p.Q1919P [37]) (Supplementary Fig. 1B), consistent with previous reports [4, 5, 13, 15]. Using the Loewe Synergy Score [38, 39], we found a synergistic interaction between olaparib and AZD6738 selectively in ATM-deficient cells, with the strongest synergy score (7.09) observed in the isogenic FaDu HNSCC *ATM*-KO cells and a modest score (3.14) in the *ATM*-mutated NCI-H23 NSCLC cells (Fig. 1ai-ii). No combination activity (synergy scores ≤0.63) was observed in *ATM* wild-type (WT) HNSCC or NSCLC cell lines (FaDu, A549, NCI-H460). Abrogated ATM signalling in response to ionising radiation (IR), assessed by auto-phosphorylated ATM (pS1981) and phospho-KAP1 (pS824), was observed in both the FaDu *ATM*-KO and NCI-H23 cells, compared with functional signalling in their WT counterpart cells (Supplementary Fig. 2), suggesting that olaparib/AZD6738 combination activity is associated with reduced ATM expression and function. Olaparib treatment also increased phospho-ATM (pS1981) in WT cells (Fig. 1b) and synergised with the ATM inhibitor (AZD0156) in all *ATM*-WT (but not ATM-deficient) cell lines (Fig. 1a), demonstrating the importance of ATM function in repairing olaparib-induced DSBs. Notably, AZD6738 did not inhibit ATM signalling at the highest doses used in this manuscript (Supplementary Fig. 3).

Combined olaparib/AZD6738 was cytotoxic in FaDu *ATM*-KO cells at various doses, whereas it was cytostatic in NCI-H23 cells (Fig. 1c), consistent with the synergy scores observed. Conversely, single-agent treatments induced only weaker cytostatic effects. Combining olaparib and AZD6738 caused a dose-dependent change in the GI_50_ values of each agent (Fig. 1d), with olaparib doses as low as 30 nM in combination with 1 μM AZD6738 causing cell kill in FaDu *ATM*-KO cells. These data suggest that in combination it may be possible to optimise lower doses of each inhibitor to achieve a greater therapeutic response in patient tumours than can be achieved by using maximum tolerated doses of either single-agent. Importantly, the highest doses (3 μM olaparib + 1 μM AZD6738) only caused moderate (39%) growth inhibition in WT cells (Fig. 1aii), suggesting a therapeutic window in ATM*-*deficient cancers. This was confirmed with a chemically diverse ATR inhibitor (VE-822) and by siRNA-mediated depletion of ATR (Supplementary Fig. 4A–D). The synergistic combination activity of AZD6738 and olaparib specifically in ATM-deficient cells indicated a biological interaction. We therefore focused on this combination for further study.

### AZD6738 abrogates the olaparib-induced DNA damage G2-M checkpoint

**Fig. 2:**
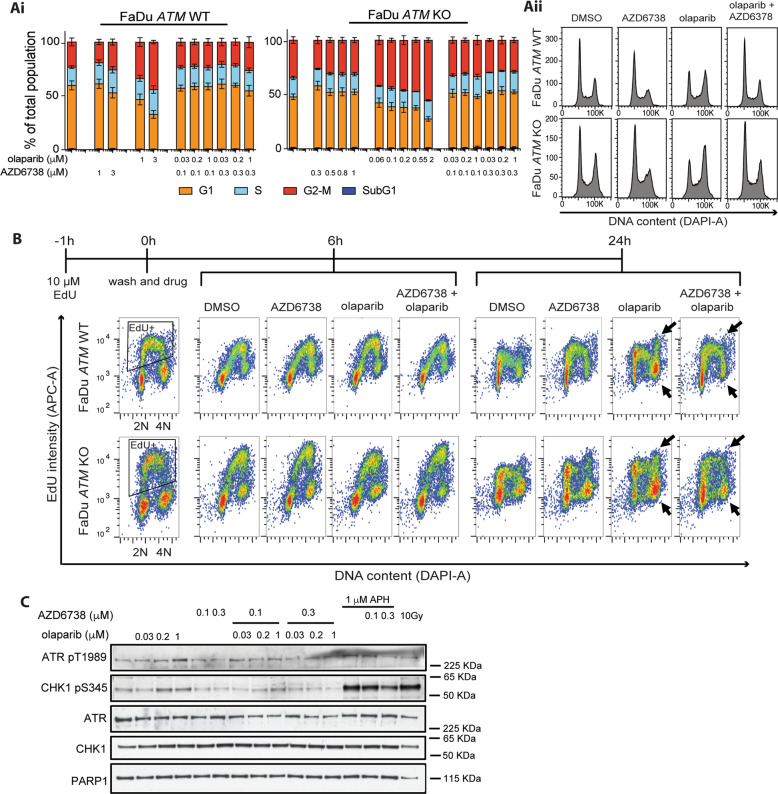
AZD6738 abrogates the olaparib-induced DNA damage G2-M checkpoint. **a** Cell cycle distributions of FaDu *ATM*-WT and KO cells following 24 h treatment with DMSO, olaparib, AZD6738 or dual olaparib/AZD6738. (i) Cell cycle distributions were determined using DAPI intensity to identify 2N (G1) and 4N (G2-M) populations via flow cytometry. Error bars = Mean ± SEM (*n* = 4). (ii) Representative cell cycle histogram profiles. **b** EdU FACS profiles after a 1 h pulse with 10 μM EdU, followed by a 6 or 24 h chase with DMSO, olaparib, AZD6738 or olaparib/AZD6738. 2N DNA content = G1; 4N DNA content = G2-M. Representative of two biological repeats. **c** Immunoblot of ATR signalling following 24 h incubation of FaDu *ATM*-WT and KO cell lines with olaparib ± AZD6738. APH (aphidicolin) and 10 Gy IR were used as positive controls.

We hypothesised that synergy between AZD6738 and olaparib could be due to ATR regulating S-phase progression and G2-M checkpoint activation in response to replication-associated olaparib-induced DNA damage. We therefore investigated the response of isogenic *ATM*-WT/KO FaDu cells following olaparib/AZD6738 combination and single-agent treatment. Single-agents were tested at comparable growth-inhibitory doses (Supplementary Fig. 5). Olaparib treatment caused a dose-dependent increase in the G2-M population at 24 h in both cell lines, whereas AZD6738 had no pronounced effect (Fig. 2ai–ii). To assess the impact of treatment in cells undergoing DNA replication, we pulse-labelled S-phase cells with EdU prior to inhibitor treatment. The G2-M population following 24 h olaparib treatment comprised both an increased number of EdU-positive cells (top arrow) and EdU-negative cells (lower arrow) compared with the DMSO-treated cells. Here, the EdU-negative cells would have transitioned through S-phase for the first time in the presence of olaparib (Fig. 2b). However, when olaparib was co-dosed with AZD6738, cells did not accumulate in G2-M and instead progressed into a second S-phase, as indicated by the reformation of an EdU-positive S-phase population at 24 h (Fig. 2a, b). Since this was observed in both WT and *ATM-*KO cells, it suggests that G2-M checkpoint engagement was primarily driven through ATR/CHK1 signalling. Correspondingly, we observed increased phosphorylation of CHK1 at S345, a site targeted by ATR, following 24 h olaparib treatment, which was abrogated by AZD6738 (Fig. 2c).

Collectively, these data show that olaparib-induced DNA damage activates the ATR-CHK1 pathway and G2-M checkpoint to allow time for DNA repair. AZD6738 abrogates this checkpoint, presumably permitting cells to undergo mitosis in the presence of DNA damage. Although this was *ATM*-status independent, we hypothesised that ATM deficiency increases the propensity for olaparib-induced DNA damage, which may account for synergy between olaparib and AZD6738 specifically in *ATM*-KO cells.

### Combined olaparib/AZD6738 enhances genome instability in *ATM-*KO cells

**Fig. 3:**
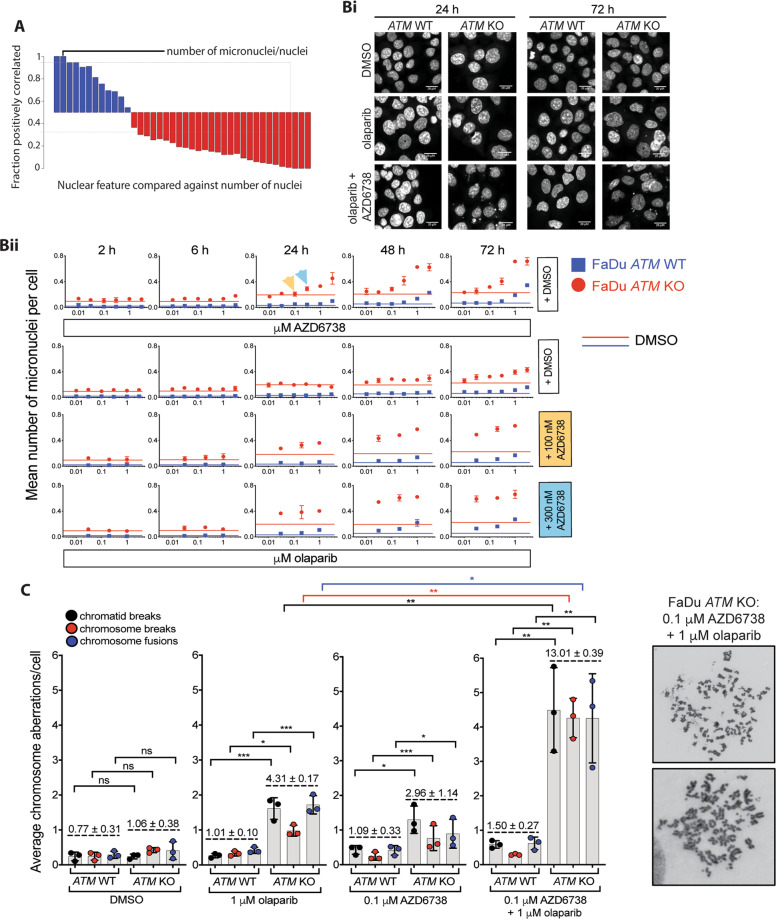
Combined PARP and ATR inhibition results in enhanced and earlier genome instability, specifically in the absence of ATM. **a** Biomarkers including mitotic index, cell cycle distribution, apoptosis, 53BP1 nuclear bodies, γH2AX and micronuclei formation were quantified in a dose- and time-dependent manner in *ATM*-WT and -KO FaDu cells. Six hundred and three nuclear features were quantified using high-throughput confocal microscopy and multiparametric image analysis (Columbus image analysis software), and correlated with the number of nuclei using a feature selection analysis strategy [62]. **b** (i) Mean number of micronuclei per cell following 2, 6, 24, 48 or 72 h single or dual olaparib and AZD6738 treatment. Micronuclei were visualised using a hoescht DNA stain and quantified using Columbus IA software (Perkin Elmer). Solid lines indicate basal levels for each individual cell line and time point. Error bars = Mean ± SD (*n* = 2). (ii) Representative images of FaDu *ATM*-WT and -KO cell lines following 24 or 72 h DMSO, olaparib, and olaparib/AZD6738 combination treatment. Images were taken at ×20 magnification (1 pixel = 0.325 μM, scale bar = 20 μM). **c** Metaphase spreads showing chromosomal aberrations in FaDu *ATM*-WT and KO cells treated with olaparib, AZD6738 or the combination for 48 h, and then arrested in metaphase of mitosis. Three types of aberrations were quantified: physical breaks in one chromosome arm (chromatid breaks), both arms (chromosome breaks), or chromosome fusions. Total number of chromosomal aberrations are indicated above the dashed line for each condition. Error bars = Mean ± SD (*n* = 3, 50 metaphase spreads/sample). *P* values calculated using a paired *t*-test *p < 0.05, **p < 0.01, ***p < 0.001. Images represent DNA fragmentation observed in some of the combination-treated FaDu *ATM-*KO cells. Representative images for each condition are shown in Supplementary Fig. 6C.

To investigate the impact of treatment and *ATM*-status on cells entering mitosis, we quantified phenotypes associated with mitotic defects using high-content immunofluorescence. This identified micronuclei number as the strongest parameter of differential response between AZD6738 and olaparib single-agent and combination treatments, and also *ATM* status (Fig. 3a). Since both ATR and ATM promote γH2AX formation it is not surprising that γH2AX foci levels, which are often used as a DNA damage biomarker [40], poorly correlated with selective efficacy in *ATM*-KO cells treated with the olaparib/AZD6738 combination (Supplementary Fig. 6Ai). This is also highlighted by a reduction in olaparib-induced γH2AX foci formation following AZD6738 co-treatment (Supplementary Fig. 6Aii). We therefore monitored the impact of olaparib and AZD6738 by assessing micronuclei formation, and observed a significant increase in basal and drug-induced micronuclei in the *ATM*-KO cells compared with the WT, alongside enhanced magnitude and kinetics of micronuclei formation following combination treatment compared with either monotherapy (Fig. 3bi–ii). Combining the lowest doses of AZD6738 (100 nM) and olaparib (30 nM) in *ATM*-KO cells induced 0.28 micronuclei/cell within 24 h, which was not achieved by either single-agent until 48 or 72 h. Conversely, in WT cells 72 h combination treatment with the highest doses (300 nM AZD6738 and 1 μM olaparib) caused only a minor increase in micronuclei that was comparable to basal levels in *ATM-*KO cells. This cannot be explained by slower cell cycle progression as the WT cells grow faster than the *ATM-*KO (Supplementary Table 1). Furthermore, micronuclei were detected before a decline in cell number, suggesting that micronuclei formation contributes to reduced cell viability in the drug-treated *ATM*-KO cells (Supplementary Fig. 6B).

To investigate the nature of genome instability detected as micronuclei, we assessed metaphase spreads 48 h after drug treatment, and observed that the AZD6738/olaparib combination synergistically induced chromatid breaks, chromosome breaks and chromosome fusions in *ATM*-KO cells, with minimal impact on WT cells (Fig. 3c, Supplementary Fig. 6C). The observed synergy is consistent with olaparib or AZD6738 single-agent treatment promoting replication-dependent chromosomal aberrations where chromatid breaks, and to some extent chromosome fusions, are more pronounced. The olaparib/AZD6738 combination significantly increased chromosomal aberrations in *ATM*-KO cells (13.0 aberrations/cell) compared with *ATM*-WT cells (1.5 aberrations/cell) and monotherapy treatment in *ATM*-KO cells (4.3 and 3.0 aberrations/cell).

PARP-inhibitor single-agent efficacy is linked to their ability to trap PARP1 onto DNA [36]. This correlates with the PARP trappers talazoparib and olaparib enhancing γH2AX and micronuclei formation, while veliparib (a weak trapper [41, 42]) did not despite equivalent near-complete inhibition of PARP catalytic activity (Supplementary Fig. 7A–C). We observed a higher synergy score between AZD6738 and talazoparib (a more potent PARP-DNA trapper) than between AZD6738 and olaparib at the dose ranges tested (Supplementary Fig. 7Di-ii). However, this combination was less selective for the *ATM*-KO cells, consistent with reports of enhanced systemic toxicity with talazoparib treatment [43]. Notably, at doses with similar single-agent efficacy, and therefore likely trapping ability, the impacts of combining AZD6738 with talazoparib versus olaparib on growth inhibition are equivalent in *ATM*-KO cells (Supplementary Fig. 7Diii). The increased synergy score therefore likely reflects the increased DNA-PARP trapping potency of talazoparib at the doses used, more than combination potential and overall cytotoxicity.

Overall, these data suggest that although the olaparib-engaged G2-M checkpoint is overridden by AZD6738 irrespective of *ATM* status, higher levels of DNA damage enter mitosis in the absence of functional ATM, as indicated by the drug-combination-dependent chromosomal fragmentation observed in various metaphase spreads (Fig. 3c). Furthermore, although both olaparib and AZD6738 exhibit monotherapy activity in *ATM*-KO cells, our data suggest that combining these agents results in a greater and faster induction of genome instability and can be achieved using lower doses.

### Combined olaparib/AZD6738 treatment causes earlier, irreversible growth inhibition and cell death in *ATM-*KO cells

**Fig. 4:**
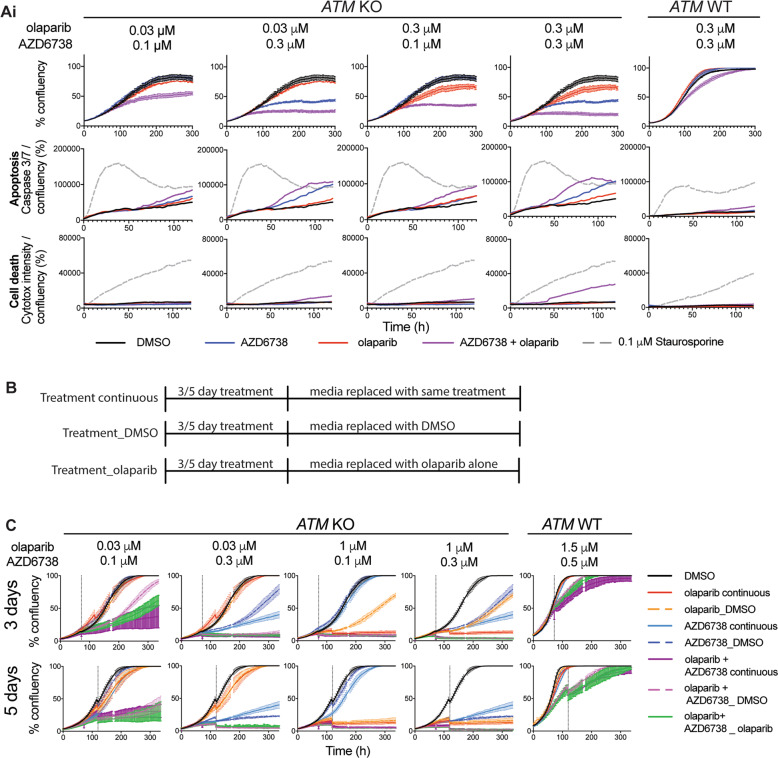
Combined olaparib and AZD6738 treatment results in earlier commitment to apoptosis in the absence of ATM. **a** Cell growth (% confluency) of cells co-stained with activated caspase 3/7 (apoptosis) and Cytotox (cell death) in FaDu *ATM*-KO and WT cells following olaparib and AZD6738 single-agent and combination treatment. Apoptosis and cell death activity is represented by mean fluorescence levels of caspase 3/7 or cytotox normalised to total cell confluency. % confluency and fluorescence intensities were calculated using incucyte ZOOM 2016A software. 0.1 μM staurosporine was used as a positive control for apoptosis. An independent repeat is shown in Supplementary Fig. 8A. **b** Schematic indicating in vitro washout dose schedules used in Figs. 4c, 5b and 6b. **c** Cell growth (% confluency) over 14 days of single and dual olaparib/AZD6738 treatment using the dose schedules outlined in Fig. 4b. Inhibitors were dosed at 0 h and the media replaced after either 3 or 5 days as indicated by the grey dashed line. Error bars = mean ± SEM (*n* = 3). Two independent biological repeats are shown in Supplementary Fig. 8Bi–ii.

We hypothesised that faster and greater micronuclei formation may cause an earlier commitment to apoptosis and cell death. We therefore measured whether growth inhibition correlated with apoptosis (active caspase 3/7) and cell death (cytotox) markers (Fig. 4a, Supplementary Fig. 8A). These experiments confirmed the selective olaparib/AZD6738 combination activity in FaDu *ATM*-KO cells, with total growth inhibition observed after 4 days compared with intermediate growth inhibition with either single-agent, and almost no impact in *ATM*-WT cells. Caspase 3/7 activity and cytotox staining were more readily detected and at earlier time points (within 36 h) following combination treatment, indicating that the reduced cell growth is predominantly due to apoptosis. The kinetics of cell death also correlated with micronuclei formation, supporting the idea that enhanced genome instability induced by co-treatment in the first 48 h, and a single cell division, is sufficient to induce apoptosis. Conversely, the later onset of caspase 3/7 activation during single-agent treatment reinforces the idea that multiple cell divisions, and/or prolonged target inhibition, are required to achieve single-agent anti-tumour efficacy. Importantly, almost no apoptotic activity was detected in the WT cells at the concentrations used, confirming their ability to tolerate the low level of micronuclei we had previously observed.

The earlier induction of micronuclei and apoptosis upon combination treatment suggests that, through synergistic activity, shorter treatment periods with lower doses could achieve similar or greater cancer-cell toxicity as long-term treatment with high-dose single agents. Current clinical monotherapy regimens for olaparib are based on continuous exposure, which is in accordance with the mechanism of cytotoxicity depending on PARP inhibition during multiple rounds of replication [44]. However, AZD6738-induced systemic toxicity may prevent continuous dosing in the clinic [45]. The opportunity to use shorter treatment periods to reduce adverse effects in patients, whilst maintaining efficacy, would provide a clinical advantage for the combination over single-agent treatment. To help guide clinical dose schedules, we compared 3- and 5-day drug washouts to continuous exposure over 2 weeks, for olaparib and AZD6738 as monotherapies and in combination. For the combination, olaparib maintenance treatment was also assessed (Fig. 4b). In *ATM*-KO cells after only 3 days, three out of four combination doses caused complete growth inhibition (Fig. 4c, Supplementary Fig. 8Bi–ii), reflecting commitment to cell death as supported by caspase 3/7 activation (Fig. 4a). After 5 days, all combination doses (including 30 nM olaparib + 100 nM AZD6738) caused irreversible growth inhibition and cell death, and olaparib maintenance was not required for durable responses. Conversely, cells treated with monotherapy regimens eventually resumed growth following inhibitor washout. In WT cells, increased doses (1.5 μM olaparib + 0.5 μM AZD6738) caused only minor growth delay. Together, these data demonstrate the potential therapeutic advantage of combining olaparib and AZD6738 in ATM-deficient tumours.

### ATM deficiency-dependent combination activity is confirmed in *TP53-*WT and *ATM*-mutant cells

**Fig. 5:**
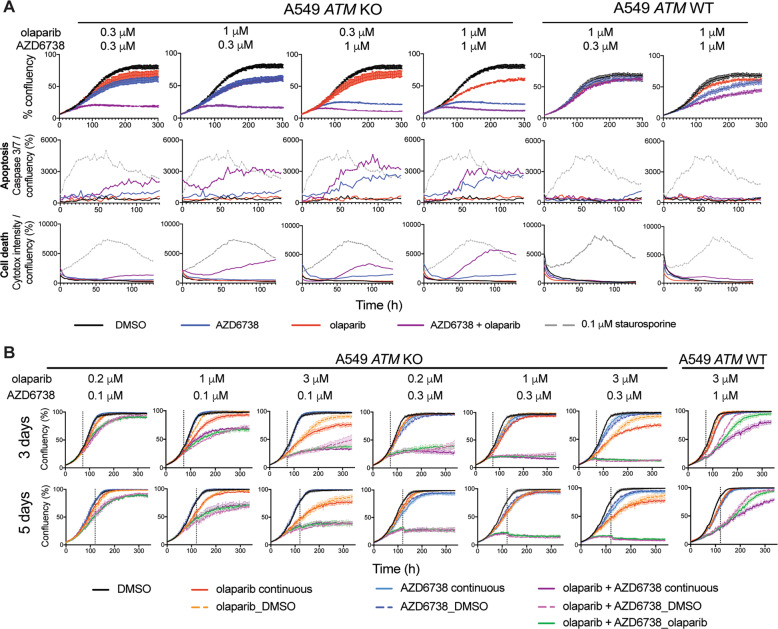
The olaparib/AZD6738 combination is still selective for ATM deficiency in *TP53-*WT A549 cells. **a** Cell growth (% confluency) of cells co-stained with activated caspase 3/7 and Cytotox in A549 *ATM*-KO and WT cells following olaparib and AZD6738 single-agent and combination treatment. 0.1 μM staurosporine was used as a positive control for apoptosis. An independent repeat is shown in Supplementary Fig. 9A. **b** Cell growth (% confluency) over 14 days single and dual olaparib/AZD6738 treatment using the dose schedules outlined in Fig. 4b. Inhibitors were dosed at 0 h and the media replaced after either 3 or 5 days as indicated by the dashed line. Error bars = mean ± SEM (*n* = 3).

Mutations in *TP53* are associated with ATR-inhibitor sensitivity in chronic lymphocytic leukaemia (CLL) [28] and in combination with DNA damaging chemo- or radiotherapy [46]. FaDu cells are *TP53-*mutant, therefore we assessed the contribution of *TP53*-status on combination efficacy using isogenic *ATM*-WT/KO *TP53*-WT A549 cells (Supplementary Fig. 2). Corroborating our findings in the FaDu cells, combined olaparib/AZD6738 repressed growth and caused apoptosis in the *ATM-*KO, but not -WT A549 cells, in a dose-dependent manner beyond what was achieved with single-agent treatments (Fig. 5a, Supplementary Fig. 9A). Correspondingly, in the *ATM*-KO cells 0.3 μM AZD6738 in combination with all olaparib doses tested completely and irreversibly inhibited cell growth within 3 days (Fig. 5b).

**Fig. 6:**
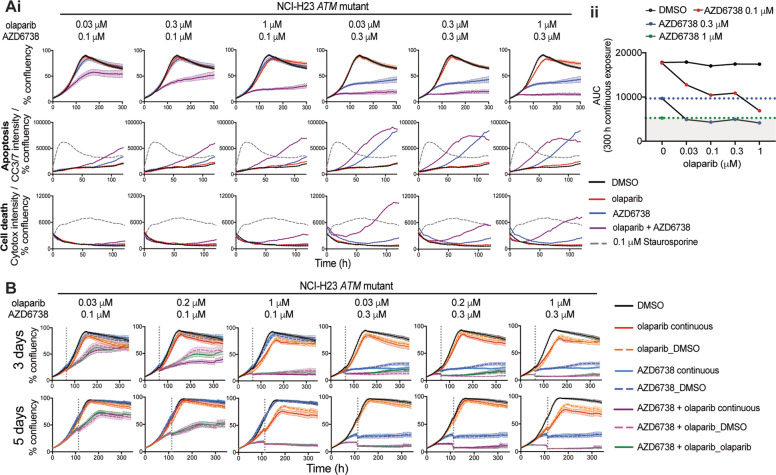
Combined olaparib/AZD6738 treatment causes cell death in *ATM*-mutant NCI-H23 lung carcinoma cells. **a** (i) Cell growth (% confluency) of cells co-stained with activated caspase 3/7 and Cytotox in NCI-H23 cells following olaparib and AZD6738 single-agent and combination treatment. 0.1 μM staurosporine was used as a positive control for apoptosis. (ii) Area under the curve (AUC) for % cell confluency over 300 h continuous treatment. The shaded area indicates cell killing greater than 1 μM AZD6738 single-agent treatment. An independent repeat is shown in Supplementary Fig. 9B. **b** Cell growth (% confluency) over 14 days single and dual olaparib/AZD6738 treatment using the dose schedules outlined in Fig. 4b. Inhibitors were dosed at 0 h and the media replaced after either 3 or 5 days as indicated by the dashed line. Error bars = mean ± SEM (*n* = 3).

*ATM*-mutated tumours demonstrate varying reductions in ATM expression and signalling, compared with total loss in KO models. We therefore tested the olaparib/AZD6738 combination activity in *ATM-*mutant NCI-H23 cells (Supplementary Fig. 2) and observed enhanced growth inhibition with earlier induction of apoptosis than with AZD6738, while olaparib monotherapy had minimal effect (Fig. 6ai, Supplementary Fig. 9B). Combining 0.3 μM AZD6738 with any olaparib dose produced similar activity to 1 μM AZD6738 alone and was sufficient to surpass the threshold for total growth inhibition caused by maximal doses of AZD6738 tested (Fig. 6aii). Importantly, 0.3 μM AZD6738 in vitro is below the maximal dose that can be achieved clinically [45], and olaparib showed minimal single-agent activity, thereby supporting the optimisation of lower drug doses in combination to achieve the same or greater endpoint. Furthermore, cells treated with 1 μM olaparib + 0.1 μM AZD6378 were unable to recover following 3 days treatment, compared with the monotherapies which induced minimal, and temporary, growth inhibition (Fig. 6b).

### Combined PARP/ATR inhibition promotes anti-tumour efficacy in xenograft and PDX models with ATM loss

**Fig. 7:**
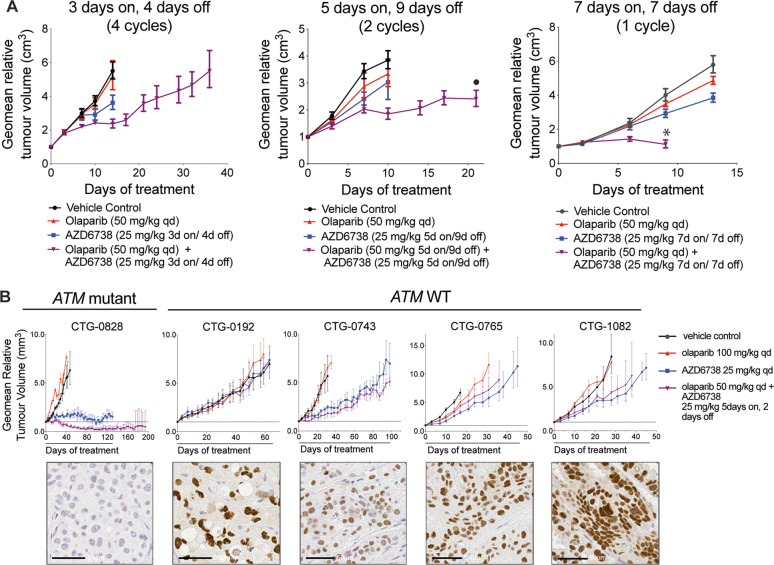
Combined PARP and ATR inhibition demonstrates anti-tumour efficacy against cell line xenograft and PDX models with ATM loss. **a** Anti-tumour effect of different schedules of AZD6738 in combination with olaparib in FaDu *ATM*-KO xenografts. Olaparib was dosed 1 h after AZD6738. All graphs represent geometric mean ± SEM, SCID mice (3 days on 4 days off *n* = 10, 5 days on 9 days off *n* = 9, 7 days on 7 days off *n* = 15). Corresponding mouse body weights and individual tumour spider plots can be found in Supplementary Fig. 10A, B. *Group stopped early due to body weight loss. •Group stopped early due to frequent tumour ulcerations impacting on animal well-being. **b** AZD6738 in combination with olaparib induces tumour regression in *ATM*-mutant CTG-0828, but not *ATM*-WT PDX models (nude mice, vehicle *n* = 5, treatment *n* = 3). Treatments were given for 130 days, or until animals were taken off study. Images show IHC staining for ATM expression (scale bar = 50 μM). All graphs represent geometric mean ± SEM. Corresponding mouse body weights and individual tumour spider plots can be found in Supplementary Fig. 10E, F.

Finally, we assessed the impact of combined olaparib/AZD6738 treatment on ATM-deficient tumours in vivo. FaDu *ATM*-KO cells were grafted into mice and intermittently treated with AZD6738 and olaparib single-agents or in combination for 3, 5 or 7 consecutive days (Fig. 7a, Supplementary Fig. 10A, B). With all schedules, combined treatment reduced tumour growth, whereas single-agent treatments provided little benefit over the control. These data highlight the importance of dose scheduling combination treatments to balance efficacy and tolerability, and suggest tumour growth inhibition can be achieved with as little as a 3 or 5-day treatment window [45, 47]. Note that due to frequent ulcerations of the FaDu *ATM*-KO xenograft model affecting animal well-being, only a short-term (21 days) 5 days on 9 days off olaparib/AZD6738 combination regimen could be performed. Furthermore, we treated a panel of PDX models, tested for ATM expression by IHC, with olaparib and AZD6738 alone or in combination (Supplementary Fig. 10C, D). Combination activity caused tumour regressions only in the ATM-deficient CTG-0828 model, with near-total loss of protein expression. Minimal to no combination activity was observed in the *ATM-*WT models (Fig. 7b, Supplementary Fig. 10E, F). In the CTG-0828 model, 130 days daily dosing of AZD6738 caused complete growth inhibition, whereas the combination caused tumour regressions that were maintained for 64 days after treatment was stopped, in 2 out of 3 animals (Supplementary Fig. 10F). Despite observing combination activity in both ATM-deficient in vivo models, tumour regressions were only observed in the CTG-0828 PDX. Assessing pharmacodynamic biomarkers for target inhibition (PARylation, RPA32 pS4/8 and γH2AX) did not explain these differences (Supplementary Fig. 11). These are instead likely due to variations in dosing schedules and the substantial differences in treatment times between the models. Note that treatment using an intermittent ‘5 day on’ schedule in the FaDu *ATM*-KO model was stopped after 21 days due to tumour ulcerations, compared with 130-day treatment in the CTG-0828 model, and that tumour regressions of the CTG-0828 PDX were only detected after 20 days. We therefore speculate that continued intermittent combination treatment of the FaDu *ATM-*KO xenograft may also induce tumour regression beyond the synergistic growth inhibition observed in the study-period tested. Together the in vitro and in vivo data demonstrate pre-clinical synergy between olaparib and AZD6738 in ATM-deficient tumours and provide a mechanistic rationale for clinically targeting tumours with *ATM*-inactivating mutations across multiple tissue types.

## Discussion

Olaparib (Lynparza) is a first-in-class PARP inhibitor approved for patients with advanced ovarian, breast and pancreatic cancer, particularly those with BRCA1/2 deficiencies. This study assessed new clinical opportunities for olaparib by combining treatment with other DDR inhibitors across a cell line panel comprising aberrations in key tumour suppressor, DDR and oncogenic genes. We observed that combining olaparib with the ATR-inhibitor AZD6738 was synergistic and cytotoxic in ATM-deficient cells, but not in ATM-proficient cells. Combined PARP- and ATR-inhibitor treatment has previously been investigated in BRCA*-*deficient backgrounds [32, 33, 48], including olaparib-resistant models. Here, we demonstrate that the combination therapy could be expanded to treat ATM-deficient cancers with the complete loss of protein or function.

Mechanistically, olaparib treatment activated the G2-M checkpoint in a manner that was abrogated by AZD6738, consistent with published data [33, 49]. This was *ATM-*status independent and correlated with ATR-dependent CHK1 phosphorylation (pS345). These data support ATR being the primary kinase that initiates the cell cycle checkpoint in response to olaparib treatment, and is consistent with ATR being important in the context of olaparib-induced replication-dependent DNA damage through its role in replication fork stabilisation and restart [23]. Although the olaparib-induced G2-M checkpoint was abrogated by AZD6738 independently of *ATM* status, we detected greater and earlier formation of micronuclei upon olaparib/AZD6738 combination treatment, specifically in *ATM*-KO cells. A synergistic increase in chromosomal aberrations in *ATM*-KO cells, detected by metaphase spread analysis, confirmed increased transmission of DNA breaks into mitosis. These data suggest that ATM is critical for maintaining genome stability in response to olaparib/AZD6738 combination-induced DNA damage in S-phase. The olaparib-induced genome instability in *ATM-*KO cells can be largely explained by ATM counteracting toxic end-joining to allow faithful repair of seDSBs [17]. Low doses of AZD6738 also generated replication-associated chromosomal aberrations in *ATM*-KO cells, although ATM’s role here is less clear. Previous reports show that ATR and ATM could share hundreds of targets [18, 20]. Redundant functions between these kinases may therefore become evident when both are inactivated [50–52]. Supporting ATM’s role in response to PARP/ATR inhibition, olaparib and AZD6738 reportedly promote RAD50 phosphorylation in ATM-functional in vivo models, but not when ATM is absent [53]. ATR is also suggested to directly promote HRR through stimulating the BRCA1–PALB2 interaction and PALB2 localisation after DNA damage [54]. Further investigations are required to assess which of the abovementioned hypotheses contribute to the sensitivity of ATM-deficient cells to AZD6738. Nevertheless, the synergistic phenotypes observed upon combination treatment of ATM-deficient cells supports the idea that olaparib and AZD6738 impact on similar biological processes to drive genome instability and cell death. Based on known ATR functions, AZD6738 likely mechanistically synergises with olaparib not only through G2-M checkpoint abrogation, but also by preventing stabilisation and restart of stalled replication forks, and by impacting HRR of collapsed replication forks. Combined AZD6738/olaparib could therefore generate additional DNA breaks which would be erroneously repaired in the absence of ATM, leading to further genome instability and cell death.

Interestingly, *ATM*-KO cells exhibited higher basal micronuclei levels, confirming that DNA damage associated with ATM-deficiency can progress into mitosis. Consequently, ATM-deficient cancers could be closer to mitotic catastrophe and thus more vulnerable to exogenous DNA damage. Endogenous micronuclei levels could therefore represent a predictive biomarker of response to agents that promote genome instability, such as ATR and PARP inhibitors, and warrants further investigation.

Key to clinical drug development is the assessment of efficacy, safety and tolerability. Maximum tolerated doses for olaparib and AZD6738 single-agent treatments have been established through clinical trials, wherein haematological toxicity was dose- and schedule-limiting [55, 56]. Given the mechanistic synergy that we observed between olaparib and AZD6738, we reasoned that the combination therapy in ATM-deficient patients may provide a better therapeutic window. Our studies provide several lines of pre-clinical evidence supporting this. Most notably, the increased and earlier formation of micronuclei (within 24 h) at sub-maximal doses of olaparib and AZD6738 in combination correlates with enhanced growth inhibition and earlier onset of apoptosis specifically in ATM-deficient cells. Together with G2-M checkpoint abrogation by AZD6738, these data imply that a single round of replication and aberrant mitosis is sufficient to induce cell death in vitro, thus providing a mechanistic rationale for optimising shorter combination treatment schedules for improved clinical tolerability and efficacy. Conversely, 72 h olaparib single-agent treatment generated low levels of micronuclei, which is consistent with olaparib being dosed continuously in the clinic to accumulate sufficient DNA damage through multiple rounds of DNA replication and cell division. Secondly, our results showed similar levels of in vitro efficacy between short or continuous drug-combination exposures. When combined, low concentrations of olaparib and AZD6738 caused cell death within 3 or 5 days, leading to durable responses when the inhibitors were removed. Conversely, single-agent treatments at the same concentrations had either no effect on proliferation or cells recovered from the growth-inhibitory effect after drug removal, suggesting a requirement for high doses and/or prolonged treatment. Together, these findings suggest the potential to develop intermittent dosing schedules of combination therapy, with or without single-agent maintenance treatment, for improved clinical tolerability and efficacy. Furthermore, we corroborated the in vitro efficacy of AZD6738/olaparib observed in *ATM*-KO FaDu cells using ATM-deficient lung carcinoma cell lines, and in vivo xenograft and PDX ATM-deficient models. Conversely, olaparib monotherapy demonstrated variable efficacy, with little or no activity observed in either the *ATM*-mutant NCI-H23 cells or in vivo ATM-null PDX model. Alongside a previous report [49], these pre-clinical findings suggest that combined olaparib/AZD6738 treatments may provide more durable responses than single-agents in ATM-deficient tumours across multiple tissues types with varying degrees of ATM-deficiency.

*ATM* is among the most commonly aberrant genes in sporadic cancer [11, 31]. However, the mutation spectrum is broad [31] and the impact on ATM functionality, tumour behaviour and response to therapy is not fully established. For example, Phase II/III trials combining paclitaxel with olaparib in patients with advanced gastric cancers, where ATM-status was stratified by immunohistochemical assessment, revealed conflicting results regarding overall survival [57]. These findings highlight the need to define the context of ATM-deficiency and establish robust patient-selection biomarkers, to maximise the therapeutic benefit for combined olaparib/AZD6738 treatment in patients. Important insights into response rates in patients with DNA repair deficiencies (such as mono and biallelic inactivation of *ATM* or *BRCA1/2*) are anticipated from clinical studies testing monotherapy or combination treatments with PARP and ATR inhibitors (NCT02987543). Interestingly, clonal evolution has been described for haematological cancers, where *ATM/11q* deletions are among several mutations identified as sub-clonal in CLL [58, 59]. Although the impact of sub-clonality and ATM deficiency in solid tumours is less well established, once ATM deficiency is robustly clinically defined it will be important to study primary samples across various tumour types to assess the impact of clonal divergence on ATM deficiency and response.

Despite olaparib and ATR inhibitors demonstrating various degrees of monotherapy efficacy in ATM*-*deficient cancers [13–15, 27–29, 60, 61], our work highlights the importance of exploring their use in combination through the potential to optimise lower doses and shorter treatment periods due to synergistic activity. This could have multiple clinical advantages. First, single-agent systemic toxicity may prevent high-dose continuous treatment that is commonly required in vitro to achieve the same level of anti-tumour efficacy as lower-dose combination therapy. The rapid killing achieved with low-dose combination therapy should allow various dose schedules to be investigated to balance clinical efficacy with systemic toxicity. Second, our findings that combination treatment generates micronuclei within 24 h suggests that sufficient DNA damage arises during the first round of DNA replication and subsequent mitosis following drug exposure. In a heterogeneous tumour where cells have variable growth rates, combination therapy could have a major advantage over either single-agent by achieving cytotoxicity with fewer rounds of replication and without chronic target inhibition. Finally, the potential to induce equivalent or greater tumour toxicity in a shorter time frame, and with lower doses, could limit acquired resistance developing during prolonged high-dose drug exposure. Achieving a deeper and durable clinical response could also overcome innate resistance, and merits further investigation. This work therefore supports the clinical line-of-sight for the development of AZD6738 in combination with olaparib and identifies ATM deficiency as a potential patient stratification strategy.

## Materials and methods

Materials and methods can be found in the supplementary file on Oncogene's website.

## Supplementary information

Supplementary information including materials and methods

Supplementary table 1
