# Supplementary material for: Combined PARP and ATR inhibition potentiates genome instability and cell death in ATM-deficient cancer cells
Source: Oncogene. 2020 May 23;39(25):4869–83. doi: 10.1038/s41388-020-1328-y (PMC7299845; doi:10.1038/s41388-020-1328-y)
Supplement: Supplementary file 1 — Supplementary information including materials and methods [file 41388_2020_1328_MOESM1_ESM.pdf]

Supplementary information for

# Combined PARP and ATR inhibition potentiates genome instability and cell death in ATM-deficient cancer cells.

Rebecca L Lloyd, Paul WG Wijnhoven, Antonio Ramos-Montoya, Zena Wilson, Giuditta Illuzzi, Katarzyna Falenta, Gemma N Jones, Neil James, Christophe D Chabbert, Jonathan Stott, Emma Dean, Alan Lau, Lucy A Young.

**Corresponding author**

Lucy A Young

Email: Lucy.Young@astrazeneca.com

**This PDF file includes:**

Supplementary figures 1-11 and figure legends

Supplementary table 1 legend

Supplementary materials and methods

References for supplementary materials and methods

Supplementary table 1 is provided separately

## Supplementary figures and figure legends

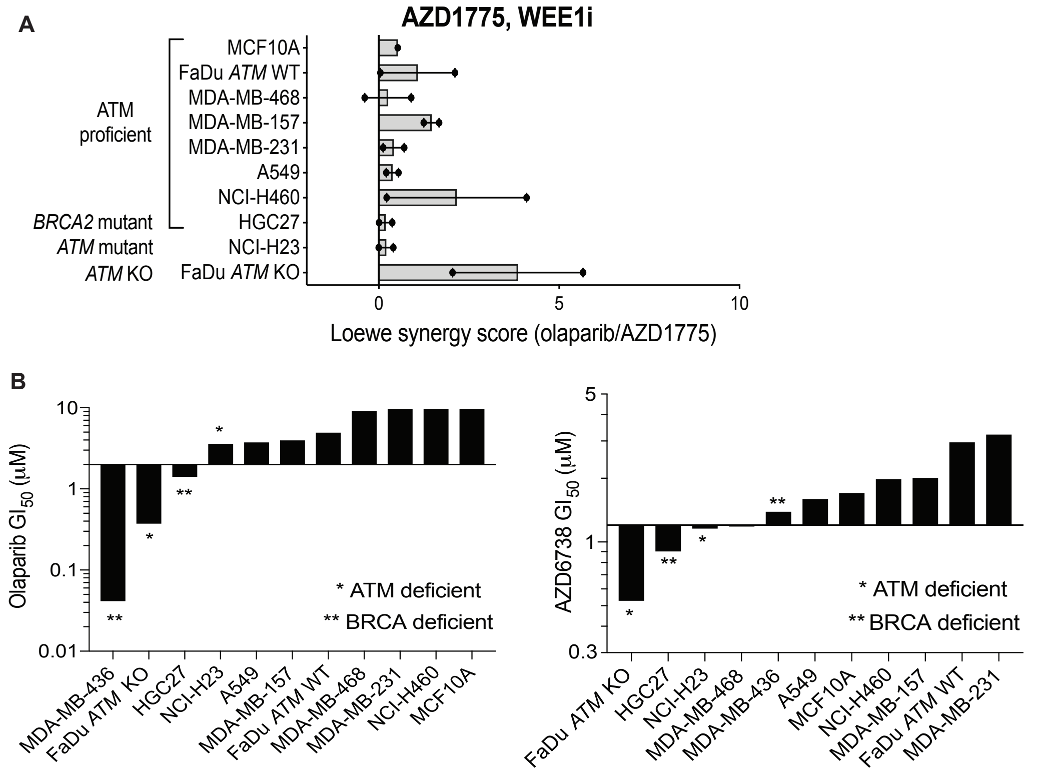


**Supplementary figure 1:**

A) Loewe synergy scores for olaparib in combination with WEE1 inhibitor AZD1775 across different cell lines. Cell viability was measured by a sytox green live-dead assay, and synergy scores calculated using the Loewe additivity model. Higher positive scores indicate greater synergistic activity. Error bars = Mean ± S.E.M (n=2).

B) $GI_{50}$ values for olaparib and AZD6738 across a select cell line panel, calculated from a 10-point dose response curve averaged across two biological replicates. ATM-deficient (*) and BRCA-deficient (**) cell lines are highlighted.

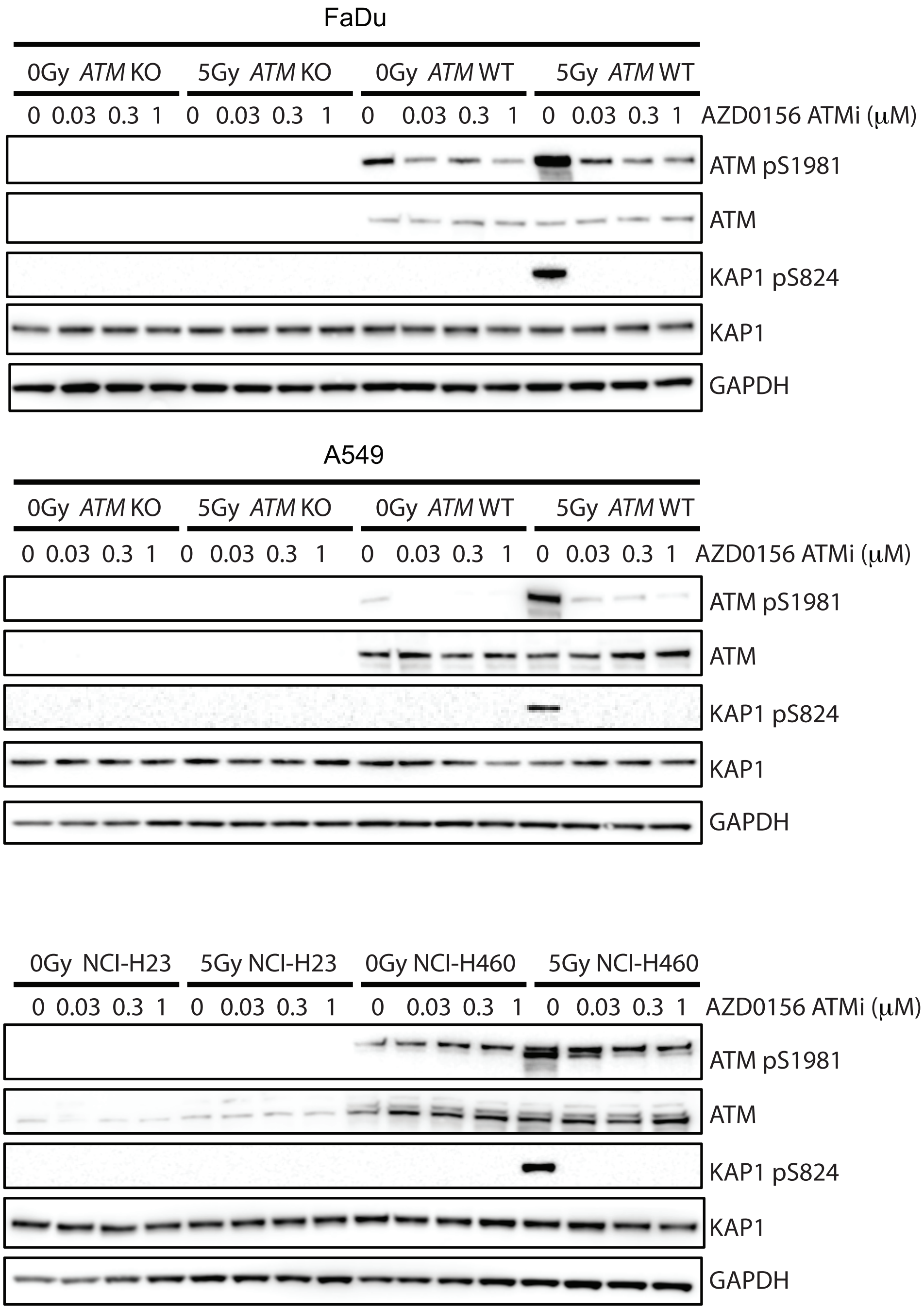


**Supplementary figure 2:** Immunoblot of ATM protein levels and signalling via ATM pS1981 and KAP1 pS824 in paired isogenic FaDu and A549 *ATM*-WT and -KO, NCI-H460 (*ATM* WT) and NCI-H23 (*ATM* mis-sense mutant) cells. 5 Gy IR was used to induce DNA damage 30 min prior to lysis, and ATM signalling was inhibited by 2h pre-incubation with ATMi AZD0156 as indicated.

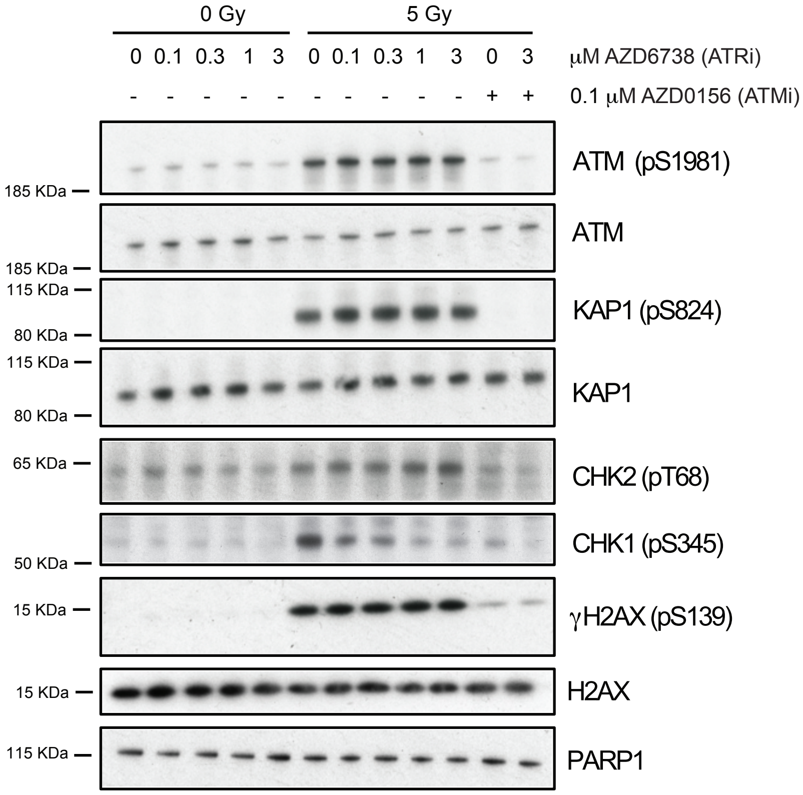


**Supplementary figure 3:** Immunoblot of ATM signalling via ATM pS1981, KAP1 pS824 and CHK2 pT68 in response to 5 Gy IR, in presence or absence of AZD6738. AZD6738 target inhibition is shown by decreasing CHK1 pS345. Cells were pre-incubated with AZD0156 and/or AZD6738 as indicated for 1h prior to irradiation and lysed 30 min later.

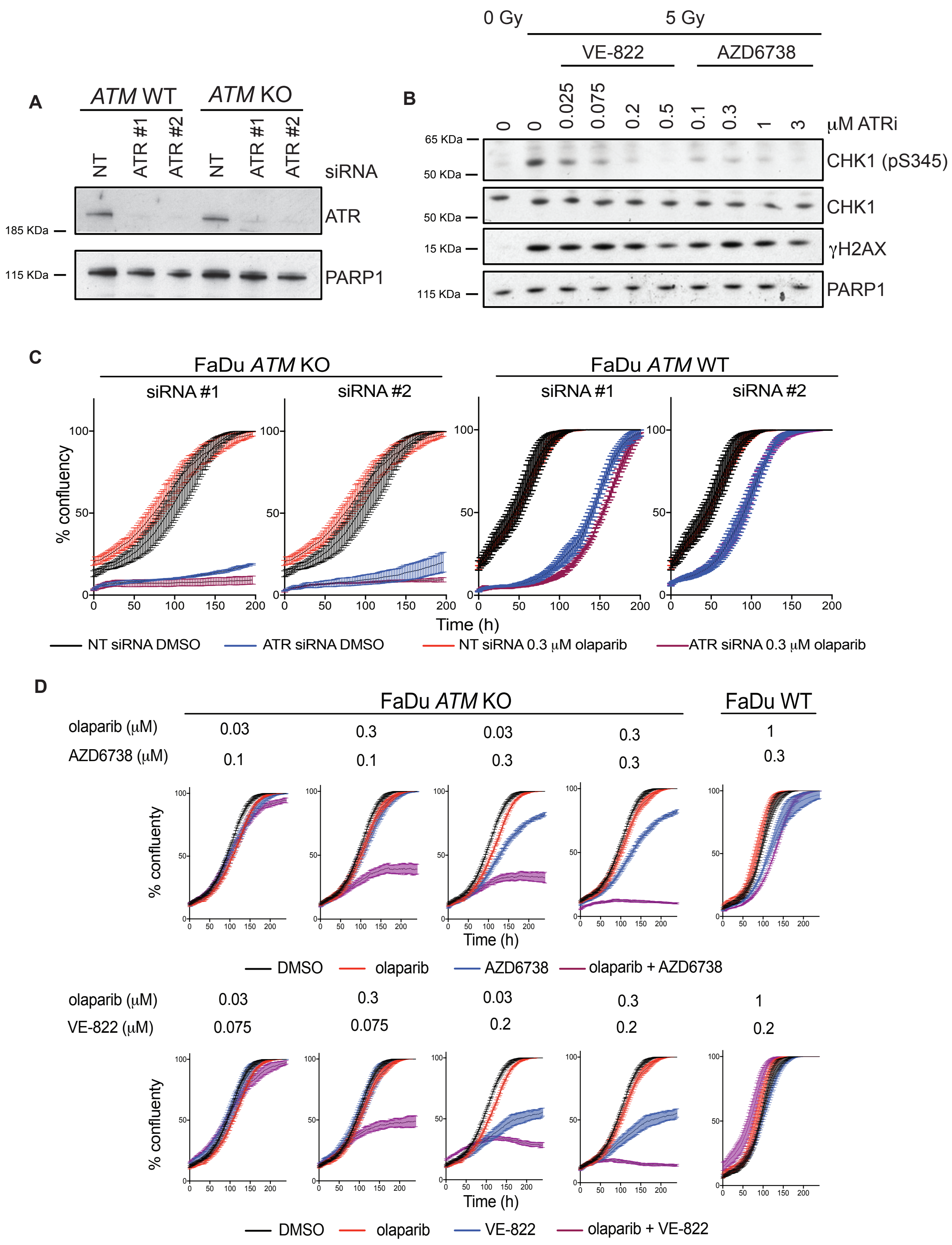


**Supplementary figure 4:**

A) Immunoblot for ATR protein levels 48h after siRNA depletion in FaDu *ATM*-WT and KO cells.

B) Immunoblot for ATR signalling via CHK1 pS345 and γH2AX in FaDu *ATM*-WT cells. Cells were pre-treated with VE-822 or AZD6738 ATR inhibitors for 1h prior to 5 Gy IR, and protein lysates collected 30 min later.

C) Cell growth (% confluency) over 9 days of FaDu *ATM*-WT and KO cells following siRNA-mediated depletion of ATR, continuously treated with or without 0.3 μM olaparib. Representative of two independent repeats. Error bars = mean ± S.E.M of three technical replicates.

D) Cell growth (% confluency) over 10 days of FaDu *ATM*-WT and KO cells in response to continuous treatment with olaparib ± AZD6738 or VE-822 (ATR inhibitors). AZD6738 and VE-822 were used at doses that gave comparable target inhibition (supplementary figure 4B). Representative of two independent repeats. Error bars = mean ± S.E.M of three technical replicates.

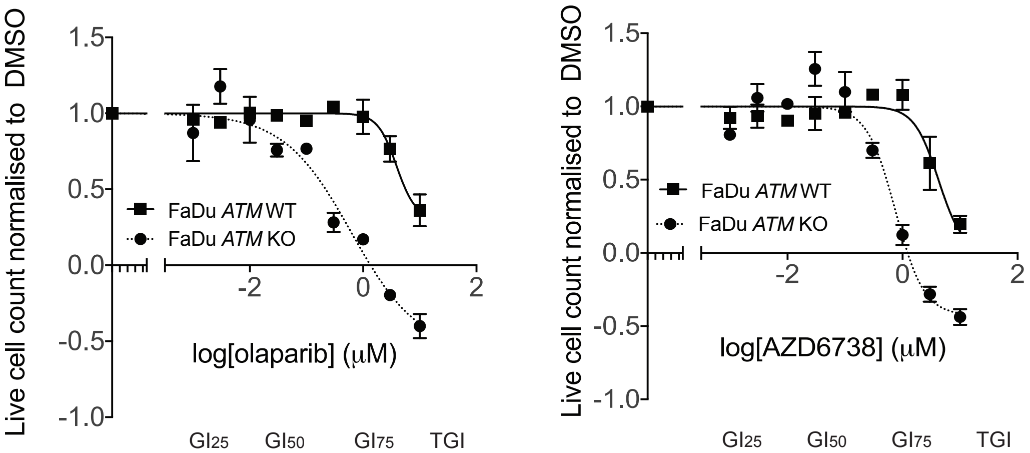


| | $GI_{25}$ | $GI_{50}$ | $GI_{75}$ | TGI |
|---|---|---|---|---|
| FaDu *ATM* WT | ~1 μM | ~3 μM | | |
| FaDu *ATM* KO | 60 nM | 200 nM | 550 nM | ~2 μM |

| | $GI_{25}$ | $GI_{50}$ | $GI_{75}$ | TGI |
|---|---|---|---|---|
| FaDu *ATM* WT | ~1 μM | 3 μM | | |
| FaDu *ATM* KO | 300nM | 500 nM | 800 nM | ~1μM |

**Supplementary figure 5:** Growth inhibition curves with calculated $GI_{25/50/75}$ and TGI (total growth inhibition, $GI_{100}$) doses for olaparib and AZD6738 in FaDu *ATM*-WT and -KO cell lines. Error bars = mean ± S.D (n=2). GI values were calculated from a 10-point dose response curve averaged across both biological replicates.

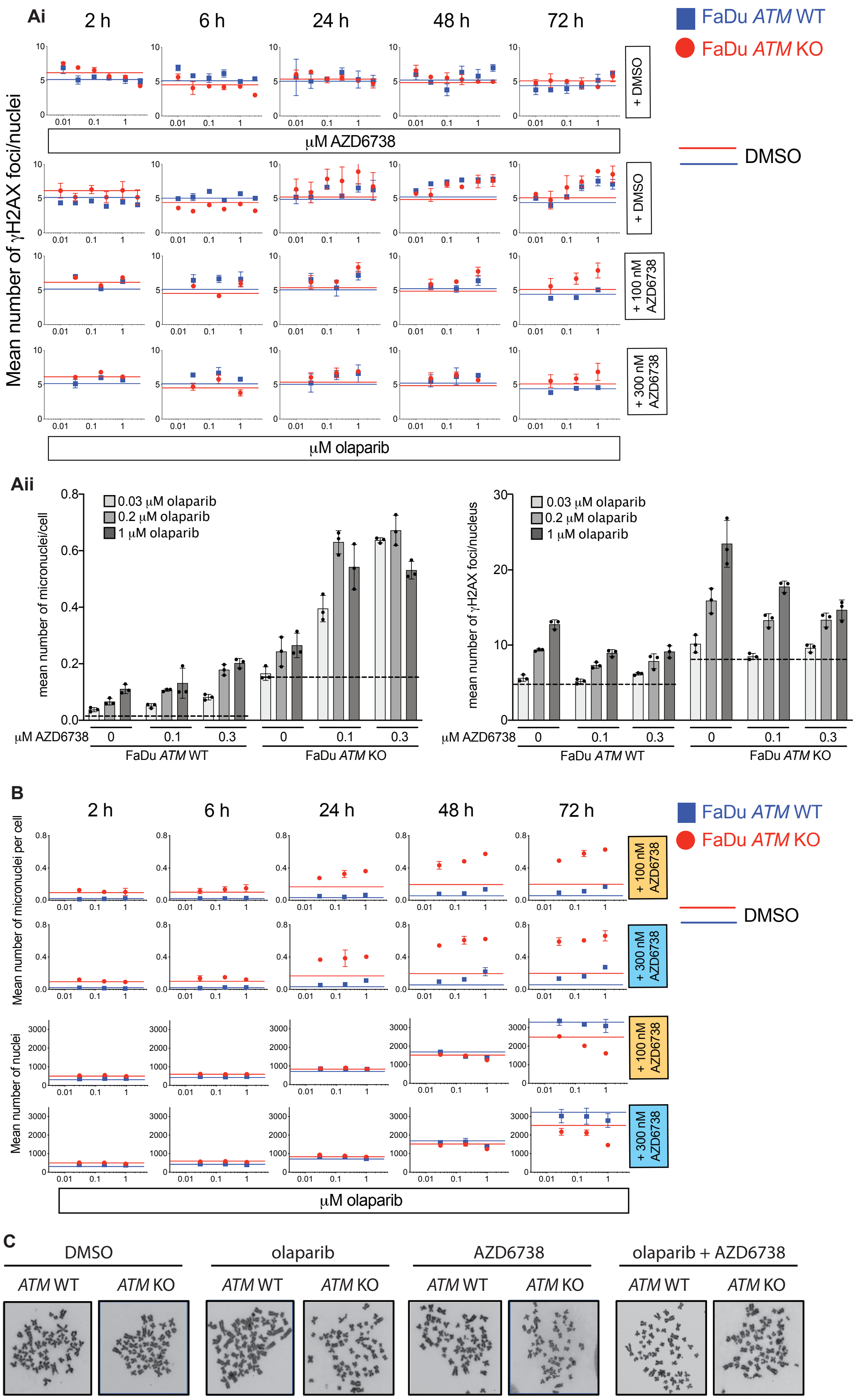
Ai
2 h
6 h
24 h
48 h
72 h
FaDu ATM WT
FaDu ATM KO
DMSO
Mean number of γH2AX foci/nuclei
+ DMSO
μM AZD6738
+ 100 nM AZD6738
+ 300 nM AZD6738
μM olaparib
Aii
0.03 μM olaparib
0.2 μM olaparib
1 μM olaparib
mean number of micronuclei/cell
mean number of γH2AX foci/nucleus
μM AZD6738
FaDu ATM WT
FaDu ATM KO
B
Mean number of micronuclei per cell
Mean number of nuclei
C
DMSO
olaparib
AZD6738
olaparib + AZD6738
ATM WT
ATM KO

**Supplementary figure 6:**

A) Mean number of γH2AX foci/nuclei following 2, 6, 24, 48 or 72h single or dual olaparib and AZD6738 treatment in FaDu *ATM*-WT and -KO cells. Foci were quantified using Columbus IA software (Perkin Elmer). Solid lines indicate basal levels for each individual cell line and time point. Error bars = Mean ± S.E.M (n=2). ii) independent comparison of mean number of micronuclei/cell and γH2AX foci/nuclei following 72h olaparib treatment ± AZD6738. Dotted lines indicate DMSO levels for each individual cell line. Error bars = Mean ± S.D (n=3).

B) Mean number of micronuclei per cell (from figure 3B) alongside cell count. Analysis was performed using Columbus IA software. Solid lines indicate basal levels for each individual cell line and time point. Error bars = Mean ± S.D (n=2).

C) Representative images of metaphase spreads quantified in figure 3C.

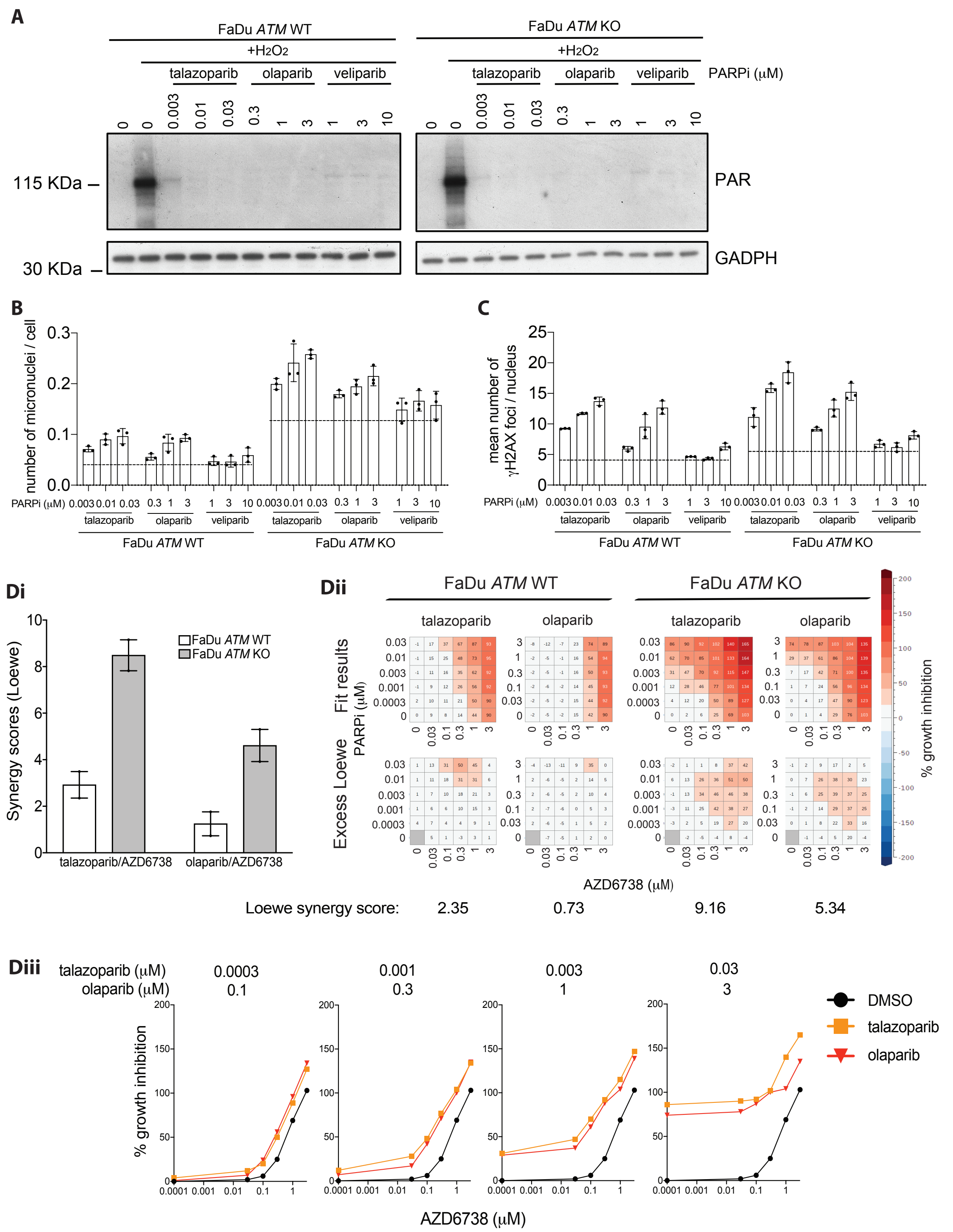


**Supplementary figure 7:**

A) Immunoblot of PARylation following 10 min 10 mM $H_2O_2$ treatment ± 90 min pre-treatment with increasing doses of olaparib, talazoparib and veliparib.

B) Mean number of micronuclei/cell in FaDu *ATM*-KO and WT cells following 72h treatment with olaparib, talazoparib or veliparib at doses that inhibit PARylation. Dotted lines indicate DMSO levels for each individual cell line. Error bars = mean ± S.D (n=3).

C) Mean number of γH2AX foci/nuclei in FaDu *ATM*-KO and WT cells following 72h treatment with olaparib, talazoparib or veliparib at doses that inhibit PARylation. Dotted lines indicate DMSO levels for each individual cell line. Error bars = mean ± S.D (n=3).

D) i) Loewe synergy scores for olaparib and talazoparib in combination with AZD6738 in *ATM*-KO and WT FaDu cells. Cell viability was measured by a CellTiter-Glo assay, and synergy scores were calculated using the Loewe additivity model. Higher positive scores indicate greater synergistic activity. Error bars = Mean ± S.E.M (n=2). ii) Representative 6x6 synergy matrix heatmaps. 'Fitted results' represent the growth inhibitory (0-100) and cytotoxic activity (100-200) based on curves fitted to the raw viability values. 'Loewe excess' represents the calculated excess activity above that expected from an additive combination, based on the Loewe additivity model. Loewe synergy scores are shown below the heatmaps. iii) Representative % growth inhibition curves for FaDu *ATM*-KO cells following combination treatment of olaparib or talazoparib with increasing doses of AZD6738. Growth curves are clustered by doses of olaparib and talazoparib that show similar single-agent efficacy. Cytostatic effects are observed in the 0-100% range and cytotoxic effects between 100-200%.

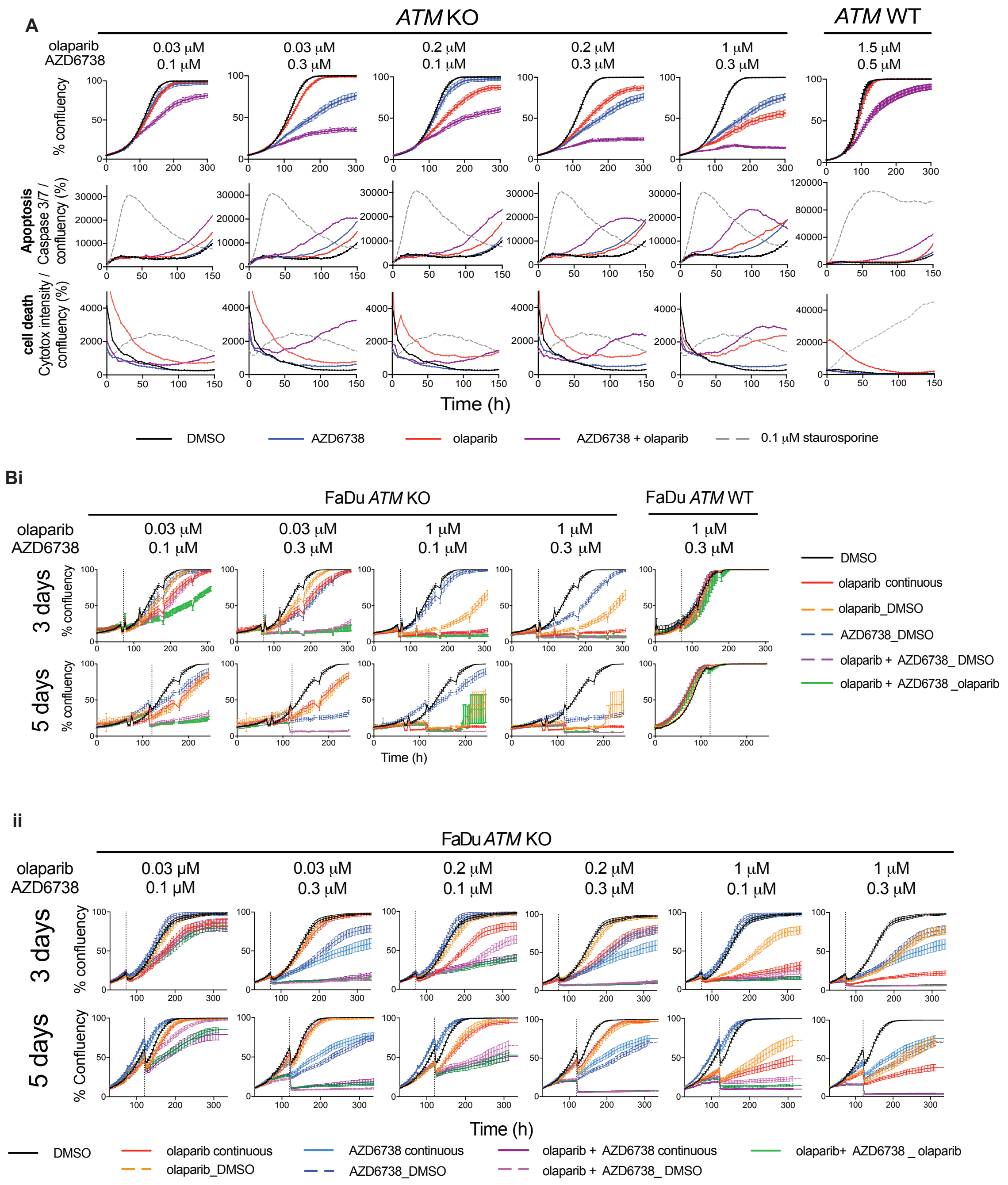


**Supplementary figure 8:**

A) Additional repeat of olaparib/AZD6738-treated FaDu cell confluency co-stained with markers of apoptosis (caspase 3/7) and cell death (cytotox). Related to figure 4A.

B) Additional repeats of olaparib/AZD6738-treated FaDu washout experiments at different drug doses. Related to figure 4C. Error bars = mean ± S.E.M.

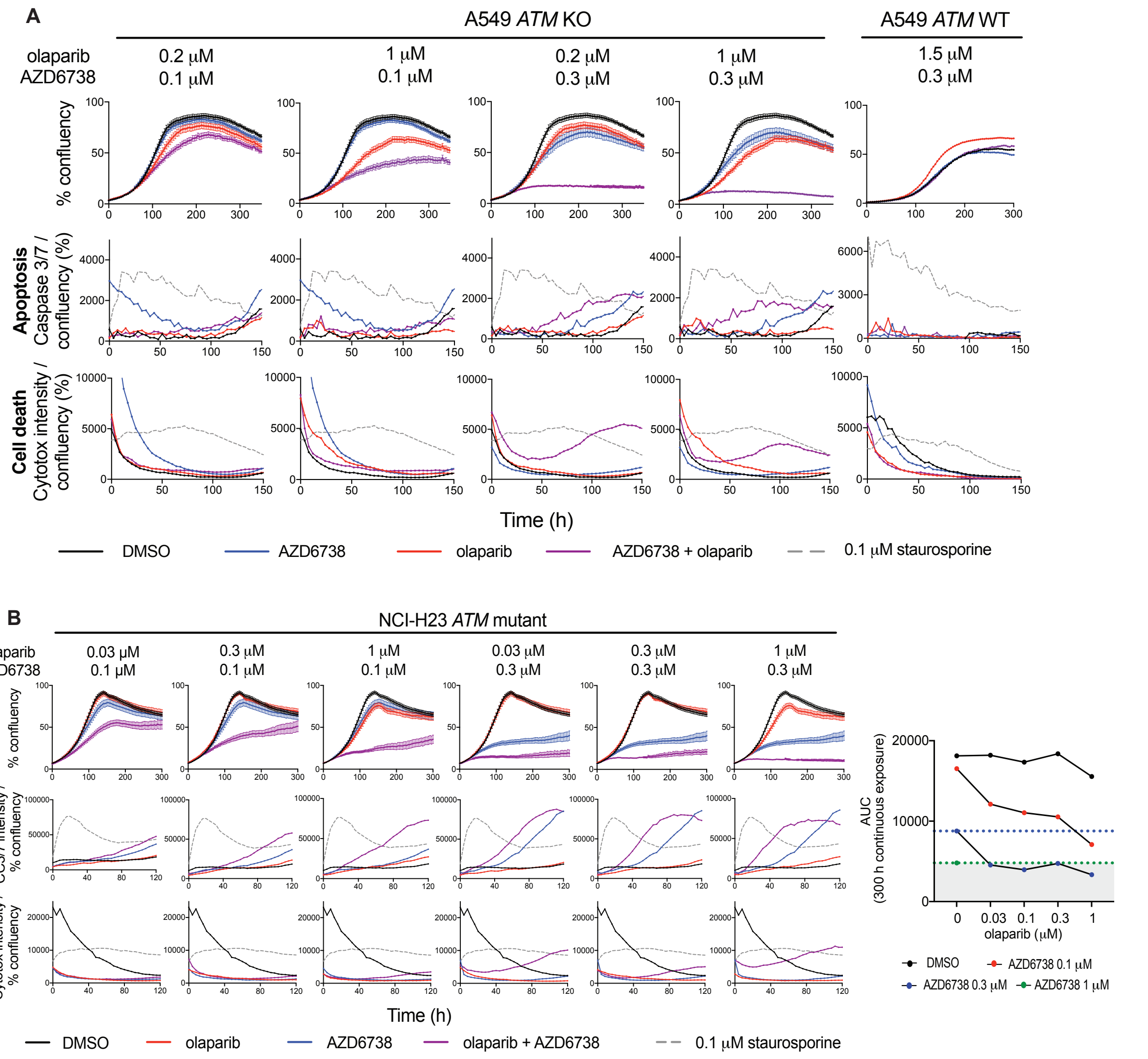


**Supplementary figure 9:**

A) Additional repeat of olaparib/AZD6738-treated A549 cell confluency co-stained with markers of apoptosis (caspase 3/7) and cell death (cytotox). Related to figure 5A.

B) Additional repeat of olaparib/AZD6738-treated NCI-H23 cell confluency co-stained with markers of apoptosis (caspase 3/7) and cell death (cytotox). Related to figure 6A.

A

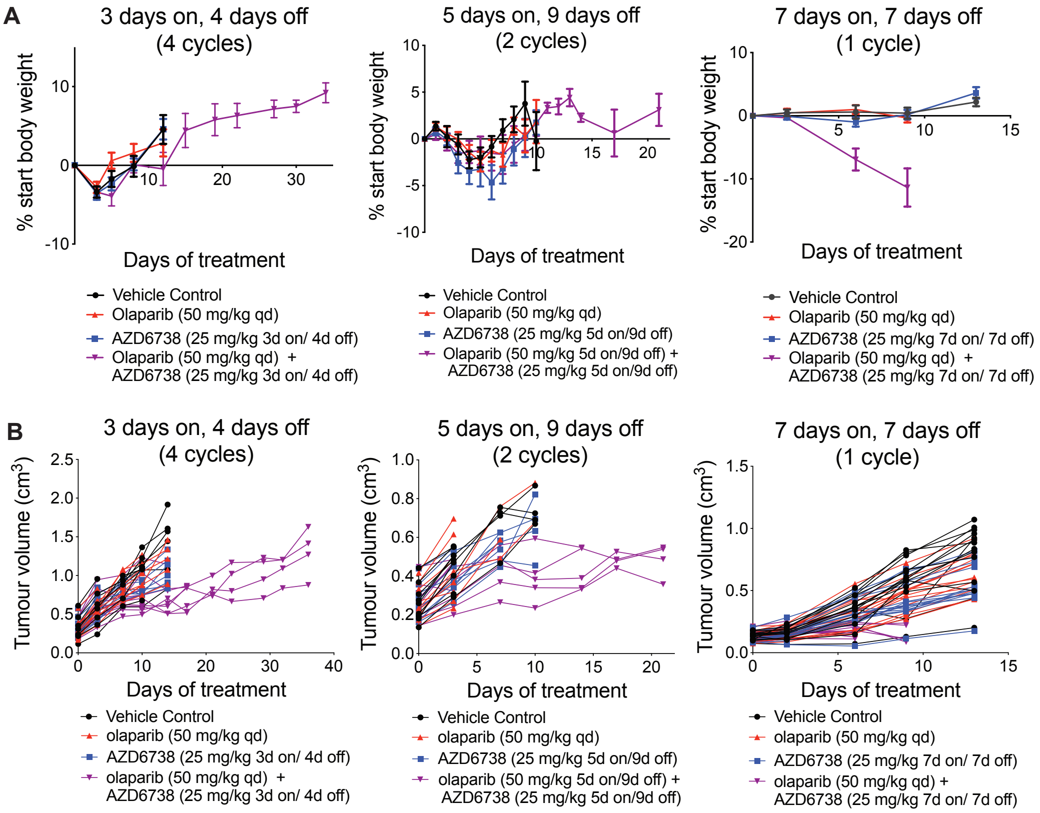


C

% maximum growth inhibition

| PDX model | CTG 0828 | CTG 0149 | CTG 1082 | CTG 0192 | CTG 0719 | CTG 1140 | CTG 0765 | CTG 0776 | CTG 0166 | CTG 1130 | LU 6439 | CTG 0198 | LU 6802 | CTG 0743 | LU 6471 | CTG 0977 | LU 6402 |
|---|---|---|---|---|---|---|---|---|---|---|---|---|---|---|---|---|---|
| AZD6738/olaparib | 163 | 61 | 55 | 0 | 50 | 51 | 44 | 0 | 77 | 37 | 25 | 73 | 0 | 88 | 0 | 69 | 38 |
| AZD6738 | 89 | 0 | 67 | 0 | 39 | 71 | 61 | 49 | 55 | 50 | 0 | 49 | 0 | 75 | 25 | 0 | 0 |
| olaparib | 0 | 45 | 0 | 0 | 34 | 78 | 36 | 35 | 0 | 0 | 29 | 26 | 0 | 0 | 0 | 51 | 0 |

D

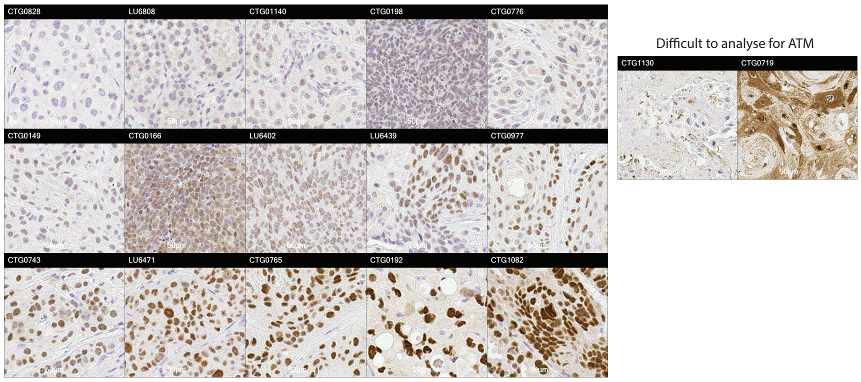


E

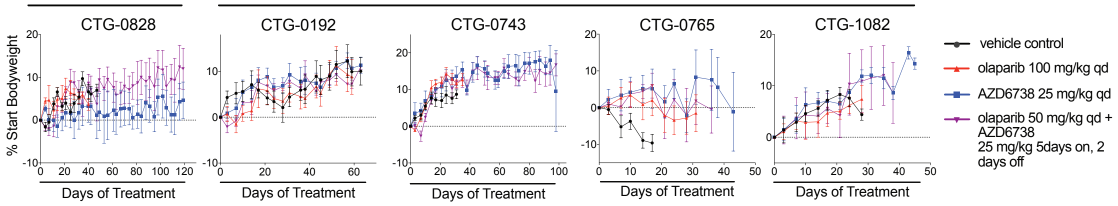


F

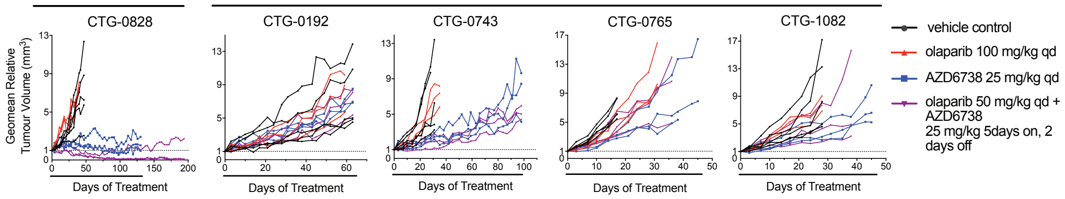

**Supplementary figure 10:**

A) Related to figures 7A. Body weights of mice used in FaDu *ATM*-KO cell line xenograft studies in response to olaparib and AZD6738 treatment. Error bars = mean ± S.E.M.

B) Related to figures 7A. Individual tumour spider plots in response to olaparib and AZD6738 treatment.

C) % maximum growth inhibition of PDX models in response to olaparib (100 mg/kg once daily), AZD6738 (25 mg/kg once daily) and dual olaparib/AZD6738 (olaparib 50 mg/kg continuous, 25 mg/kg AZD6738 5 days on 2 days off) treatment.

D) IHC staining for ATM expression across the panel of PDX models used in supplementary figure 10C. Scale bar = 50 μM.

E) Related to figure 7B. Body weights of mice used in PDX studies in response to olaparib and AZD6738 treatment. Error bars = mean ± S.E.M.

F) Related to figure 7B. Individual PDX tumour spider plots in response to olaparib and AZD6738 treatment.

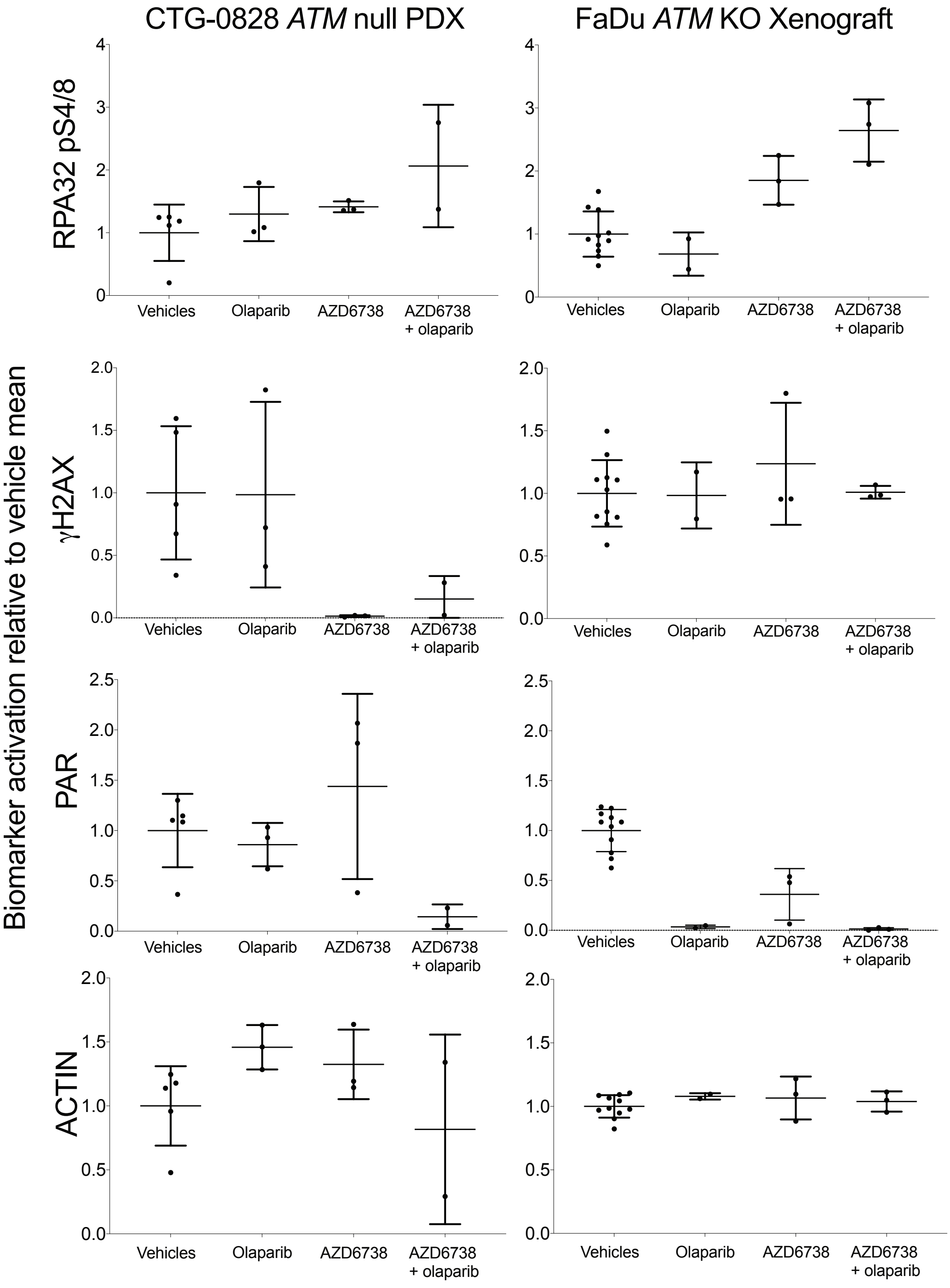


**Supplementary figure 11:** Pharmacodynamic biomarkers PAR, γH2AX and RPA32 pS4/8 in FaDu *ATM*-KO xenograft and CTG-0828 *ATM*-null PDX tumour samples after final dose of olaparib (100 mg/kg CTG-0828, 50 mg/kg FaDu *ATM*-KO), AZD6738 (25 mg/kg) or dual olaparib/AZD6738 (50 mg/kg olaparib, 25 mg/kg AZD6738). Samples were analysed via western blot and quantified using Genetools software. Error bars = mean ± S.D.

**Supplementary table 1:** Additional information on mutations, olaparib/AZD6738 $GI_{50}$ values and synergy scores, and assay set up for the panel of cells lines used in figure 1. The genomics of cell lines were acquired from canSAR (https://cansarblack.icr.ac.uk).

## Materials and methods

**Cell lines and compounds:** All cell lines were originally obtained from ATCC. FaDu and A549 *ATM*-KO cell lines were previously generated by AstraZeneca[1]. Cell line identification was validated using the CellCheck assay (IDEXX Bioanalytics, Westbrook, ME, USA). All cell lines were validated free of virus by IMPACT tests (IDEXX Bioanalytics) and validated free of *Mycoplasma* contamination using the MycoSEQ assay (Thermo Fisher Scientific, Waltham, MA, USA) or STAT-Myco assay (IDEXX Bioanalytics). All cell lines were grown in RPMI-1640 growth media (Sigma-Aldrich, St. Louis, MO, USA) supplemented with 10% fetal bovine serum (FBS) and 2 mM glutamine, apart from A549 (Ham's F-12 Nut mix (Thermo Fisher Scientific) + 10% FBS), MCF10A (MEGM + supplements as per Lonza instructions; Lonza, Basel, Switzerland) and MDA-MB-436 cells (RPMI + 10% FBS + 2 mM glutamine + 10 μg/ml insulin). AZD6738, olaparib, AZD1775 and AZD0156 were made by AstraZeneca (Cambridge, UK). Aphidicholin was obtained from Sigma (A0781). VE-822 (S7102), talazoparib (S7048) and veliparib (S1004) were obtained from Selleckchem (Houston, TX, USA). All compounds were solubilised in DMSO. To compare doses that caused equivalent responses in FaDu *ATM*-WT and KO cells, single-agents were used at their $GI_{25}$, $GI_{50}$, $GI_{75}$ and total growth inhibition (TGI, $GI_{100}$) values (supplementary figure 5). In combination, the lowest doses of both inhibitors that caused a shift in the $GI_{50}$ in *ATM*-KO cells were tested (AZD6738; 100 nM, olaparib; 30 nM), alongside intermediate and clinically relevant doses.

***In vitro* growth inhibition and cell viability assays**: Cells in 384-well plates were dosed using an Echo 555 (LabCyte, San Jose, CA, USA), and assay length normalised for doubling time (supplementary table 1). Live cell count pre- and post-treatment was determined using a Sytox Green endpoint as previously described[2], or using CellTiter-Glo as per manufacturer's instructions (Promega, Madison, WI, USA; G7570). Data was converted to 0-200% growth inhibition in Genedata Screener as previously described[3] and synergy scores were calculated by comparison to the Loewe model of additivity[4]. For both biological repeats, synergy scores and growth inhibition (GI) values were calculated from growth curves averaged from two technical replicates. Dose-

response curves were plotted in GraphPad prism V.6 using the nonlinear regression model, sigmoidal dose response, and GI values calculated using interpolated X values from these curves.

***Incucyte cell confluency, washout and apoptosis assay*:** Cells in 96-well plates were dosed using the HP D300e Digital Dispenser (HP Life Science Dispensing) and incubated with caspase 3/7 green apoptosis reagent and Cytotox red (Essen bioscience, Royston, UK) when indicated. For washout experiments, growth media was replaced at the indicated time point and re-dosed with DMSO or compound using the HP D300e Digital Dispenser. Live-cell imaging was acquired at 10x magnification at least every 6h, and cell confluency and fluorescence intensity were quantified using incucyte ZOOM 2016A software (Essen Bioscience). Cell growth was quantified using percentage phase confluency and relative apoptosis/cell death determined using fluorescence (GCU or RCU x $\mu M^2$/image) / (% confluency). AUC was calculated using GraphPad prism V.6. A representative biological replicate is shown in the main figure, and independent replicates are provided in supplementary data. Error bars for each biological replicate = mean ± S.E.M for three technical replicates.

**DsiRNA transfections:** DsiRNAs were obtained from IDT (Integrated DNA technologies, Coralville, IA, USA) and transfected using Lipofectamine RNAiMAX (Invitrogen, Waltham, MA, USA) according to manufacturer's protocol. Cells were plated for assays 48h later.
siATR #1 (hs.Ri.ATR.13.1): GUGCAUUACCUUAUACAGGAAGCTA
siATR #2 (hs.Ri.ATR.13.2): GCUGAAGGAUACCUUGAUUCUUACA
siNT (DsiRNA NC1): universal negative control by IDT

**Flow cytometry:** Cells were seeded at 100,000 cells/well in 6 well plates 18h prior to treatment. For EdU pulse chase experiments, cells were incubated with 10 μM EdU for 1h prior to washing the cells and replacing with drug-containing media. Cells were fixed in 70% ice-cold ethanol. The EdU Click-iT reaction was performed according to manufacturer's instructions (Life Technologies, Waltham, MA, USA; C10419) before staining with 1 μg/ml DAPI. Data was acquired using a FACS Aria II flow cytometer (BD bioscience, Franklin Lakes, NJ, USA) and analysed in FlowJo (Tree

Star, Inc., Ashland, OR, USA). Cell cycle profiles were analysed in 4 independent experiments, apart from 0.55 μM olaparib and 0.8 μM AZD6738 in *ATM*-KO cells, which were only analysed in 3.

**Western analysis:** Cells were lysed in RIPA buffer (Sigma-Aldrich) supplemented with protease inhibitors (Roche, Basel, Switzerland) and phosphatase inhibitors (Sigma-Aldrich). For PARylation analysis, cells were lysed directly in 2X Laemlli buffer. Equal amounts of whole cell lysates were separated on 4-12% Bis-Tris NuPAGE gels and analysed by standard immunoblotting. The following antibodies were obtained from cell signalling technologies (Danvers, MA, USA): CHK1 pS345 (CST2348, 1:1000), CHK2 pT68 (CST2661, 1:1000), PARP1 (CST9532, 1:2000), CHK1 (CST2360, 1:1000), GAPDH (CST2118, 1:2000), ATR (CST2790, 1:1000) and ATR pT1989 (CST58014, 1:1000). The following antibodies were obtained from Abcam (Cambridge, UK): ATM pS1981 (ab81292 1:1000), KAP1 pS824 (ab70369, 1:1000), KAP1 (ab10483, 1:1000), ATM (ab78, 1:2000). Antibodies against γH2AX (05-636, 1:1000) and H2AX (07-627, 1:2000) were obtained from Merck Millipore (Burlington, MA, USA) and the anti-PAR reagent (MABE1031, 1:4000) was obtained from Sigma-Aldrich. Immunoblots are representative of experiments that were performed at least twice.

**Immuno-fluorescence:** Cells in 384-well plates were dosed using the Echo 555 (Labcyte) and incubated for 2, 6, 24, 48 or 72h. Cells were fixed and permeabilised in 100% ice cold methanol for 20 min at -20°C, followed by further fixation in 4% PFA for 15 min at RT. Blocking was performed using 5% BSA in 0.05% PBS-tween and primary antibodies were incubated overnight at 4°C, followed by Alexa-Fluor secondary antibodies and Hoescht 33258 (Sigma, 2 $\mu g\ ml^{-1}$) for exactly 1 hour. The γH2AX (05-636, 1:1000) antibody was obtained from Merck Millipore, histone H3 pS10 (CST9701, 1:500) from cell signalling technologies, and 53BP1 (sc-22760, 1:1000) from santa-cruz (Dallas, TX, USA). Plates were imaged using a CV7000 spinning disc confocal microscope (Yokagowa, Tokyo, Japan) at 20X magnification with six fields of view per well, yielding over 2000 imaged cells across duplicate wells per condition. A sample size of 2000 cells was pre-determined

to allow accurate cell cycle profiling based on Hoescht intensity. For both biological replicates, data was averaged across the two technical replicates. An exposure of 250 ms and z-offset of -3 μm were used for each of the three channels; 405 nm (BP445/45), 488 nm (BP525/50), 555 nm (BP600/37) for laser excitation and camera acquisition wavelengths respectively. All data was imported into Columbus image analysis software (PerkinElmer, Waltham, MA, USA) for subsequent analysis, including the following notable parameters.

*Defining and counting nuclei:* Nuclei were defined based on hoescht intensity. Border objects were removed from analysis, alongside nuclei <30 $\mu m^2$.

*Defining and counting micronuclei:* Micronuclei were initially defined based upon the Hoechst stain using Columbus' "find micronuclei" function, and further refined based upon the following parameters: fraction of nucleus area <0.33, roundness >0.75, area >2 $\mu m^2$, fraction of nucleus intensity >0.25, intensity hoechst CV (%) <36 and distance from nucleus 0.6-10 μm.

*Counting number of γH2AX foci:* Foci were defined using Columbus' "find spots" function method B (splitting co-efficient 0.5, detection sensitivity 0.05) and spots <1$px^2$ removed.

Feature selection analysis was performed using the method described in Laufer *et al*[5], which included selecting for reproducible parameters with a Pearson correlation >0.6 between biological replicates.

**Metaphase spreads:** Metaphase spread experiments were performed as previously described in Fok *et al*, 2019[1]. Data represents biological replicates (n=3, 50 spreads/sample).

***In vivo* studies:** Immunocompromised SCID (C.B-17/IcrHan®Hsd-*Prkdcscid*) or Hsd:Athymic Nude-*Foxn1null* female mice (Envigo, Indianopolis, IN, USA) were used for tumour implantation. All mice were over 18g at the start of live-phase study. Group sizes were determined using power calculations based on internal historical growth data for individual models, and animals were randomised into treatment groups based on mean tumour volume across the groups. AZD6738 was formulated in 10% DMSO/40% Propylene Glycol and orally dosed. Olaparib was formulated in 10% DMSO/30% Kleptose and orally dosed once daily. In all combinations AZD6738 was dosed

1h after olaparib or its vehicle. Tumour volume was measured bilaterally by calliper using the formula π/6000 x length x width$^2$; animal body weight, and tumour condition was recorded twice weekly for the duration of the study. Tumour growth inhibition from start of treatment was assessed by comparison of the mean change in tumour volume for the control and treated groups, using the Mousetrap application, and represented as total growth inhibition. All *in vivo* studies complied with all relevant ethical regulations for animal testing and research, followed AstraZeneca's global bioethics policy and received ethical approval from the AstraZeneca ethical committee. Data was reported following the ARRIVE (Animal Research: Reporting In Vivo experiments) guidelines[6]. PDX studies were carried out at Champions Oncology, Inc., USA in accordance to the guidelines of the Institutional Animal Care and Use Committee (IACUC) of Champions Oncology and the USA regulatory legislation. FaDu *ATM*-KO cell line xenograft studies were conducted in the UK in accordance with UK Home Office legislation, the Animal Scientific Procedures Act 1986 and the Home Office project licences 70/8894 and P0EC1FFDF. For all animal studies, no specific blinding took place but the scientists who ran the studies were not directly involved in the associated project work or study outcomes.

**IHC analysis:** ATM IHC staining was carried out on formalin fixed paraffin embedded (FFPE) tissue sections using the Y170 antibody clone from Abcam (ab32420, 1.2 μg/ml) as described in Villaruz et al[7]. Slides were scanned at 20x magnification on the Aperio AT2 scanner (Leica, Wetzlar, Germany).

**Pharmacodynamic biomarker analysis:** Flash-frozen tumour sections were lysed in ice-cold buffer (20 mM Tris-NaCl pH 7.5, 137 mM NaCl, 10% glycerol, 50 mM NaF, 1 mM $Na_3VO_4$) supplemented with Protease complete inhibitor tablet (Roche 1836145), benzonase (1 μl/ 5ml, sigma E1014-5KU), phosphatase inhibitor cocktails (Sigma P0044 and P5726) and protease inhibitor cocktail (Sigma P8340). Samples were then homogenised and sonicated prior to addition of SDS (1% final) and NP40 (1% final). Samples were centrifuged and supernatants collected. 40 μg protein was separated on 4-12% Bis-Tris or 3-8% Tris-acetate NuPAGE gels and analysed by

standard immunoblotting. γH2AX (CST2577, 1:1000) and β-actin (CST4970,1:1000) antibodies were obtained from cell signalling technologies, RPA32 pS4/8 (A300-245A, 1:1000) from Bethyl laboratories (Montgomery, TX, USA) and PAR (4336) from Trevigen (Gaithersburg, MD, USA). Biomarker signals were quantified using Genetools software.

**Statistical analysis:** Student's paired one-tailed t-test analysis assuming unequal variances were performed to determine statistical significance in replicate comparisons. QQ-plots and the Shapiro-Wilk test showed that the samples were normally distributed. Variances were not comparable between groups as seen by the graphs and Bartlett's test. A $p$-value $< 0.05$ was deemed statistically significant.

## References


1 Fok JHL, Ramos-Montoya A, Vazquez-Chantada M, Wijnhoven PWG, Follia V, James N *et al.* AZD7648 is a potent and selective DNA-PK inhibitor that enhances radiation, chemotherapy and olaparib activity. *Nat Commun* 2019; **10**: 5065.

2 Davies BR, Greenwood H, Dudley P, Crafter C, Yu D-H, Zhang J *et al.* Preclinical Pharmacology of AZD5363, an Inhibitor of AKT: Pharmacodynamics, Antitumor Activity, and Correlation of Monotherapy Activity with Genetic Background. *Mol Cancer Ther* 2012; **11**: 873–887.

3 Crafter C, Vincent JP, Tang E, Dudley P, James NH, Klinowska T *et al.* Combining AZD8931, a novel EGFR/HER2/HER3 signalling inhibitor, with AZD5363 limits AKT inhibitor induced feedback and enhances antitumour efficacy in HER2-amplified breast cancer models. *Int J Oncol* 2015; **47**: 446–454.

4 Muischnek L and. Effect of combinations: mathematical basis of the problem. *Arch Exp Pathol Pharmakol* 1926; **114**: 313–326.

5 Laufer C, Fischer B, Billmann M, Huber W, Boutros M. Mapping genetic interactions in human cancer cells with RNAi and multiparametric phenotyping. *Nat Methods* 2013; **10**: 427–431.

6 Kilkenny C, Browne WJ, Cuthill IC, Emerson M, Altman DG. Improving bioscience research reporting: The arrive guidelines for reporting animal research. *PLoS Biol* 2010; **8**: e1000412.

7 Villaruz LC, Jones H, Dacic S, Abberbock S, Kurland BF, Stabile LP *et al.* ATM protein is deficient in over 40% of lung adenocarcinomas. *Oncotarget* 2016; **7**: 57714–57725.
